# Dynamical differential covariance recovers directional network structure in multiscale neural systems

**DOI:** 10.1073/pnas.2117234119

**Published:** 2022-06-09

**Authors:** Yusi Chen, Burke Q. Rosen, Terrence J. Sejnowski

**Affiliations:** ^a^Computational Neurobiology Laboratory, Salk Institute for Biological Sciences, La Jolla, CA 92037;; ^b^Section of Neurobiology, Division of Biological Sciences, University of California San Diego, La Jolla, CA 92093;; ^c^Neurosciences Graduate Program, University of California San Diego, La Jolla, CA 92093;; ^d^Institute for Neural Computation, University of California San Diego, La Jolla, CA 92093

**Keywords:** neural dynamics, functional connectivity, resting-state fMRI, dynamical differential covariance, Human Connectome Project

## Abstract

Investigating neural interactions is essential to understanding the neural basis of behavior. Many statistical methods have been used for analyzing neural activity, but estimating the direction of network interactions correctly and efficiently remains a difficult problem. Here, we derive dynamical differential covariance (DDC), a method based on dynamical network models that detects directional interactions with low bias and high noise tolerance under nonstationarity conditions. Moreover, DDC scales well with the number of recording sites and the computation required is comparable to that needed for covariance. DDC was validated and compared favorably with other methods on networks with false positive motifs and multiscale neural simulations where the ground-truth connectivity was known. When applied to recordings of resting-state functional magnetic resonance imaging (rs-fMRI), DDC consistently detected regional interactions with strong structural connectivity in over 1,000 individual subjects obtained by diffusion MRI (dMRI). DDC is a promising family of methods for estimating connectivity that can be generalized to a wide range of dynamical models and recording techniques and to other applications where system identification is needed.

## Significance

We sense, move, and think by dynamical interactions between neurons. It is now possible to simultaneously record from many individual neurons and brain regions. Methods for analyzing these large-scale recordings are needed that can reveal how the patterns of activity give rise to behavior. We developed dynamical differential covariance (DDC), an efficient, intuitive, and robust way to analyze these recordings and validated it on simulations of model neural networks where the ground truth was known. It can estimate not only the presence of a connection but also which direction the information is flowing in a network between neurons or cortical areas. We applied DDC to recordings from functional magnetic resonance imaging in humans and confirmed predicted connectivity with direct measurements.

Long-range communication pathways between brain areas carry signals that underlie a wide range of behaviors. Network neuroscience methods have been developed for estimating brain connectivity (1, 2). These methods are generally divided into structural, functional, and effective connectivity. Structural connectivity, assessed directly by tracing circuits with connectomics (3–5), provides anatomical ground truth, but the resulting static connection matrix does not by itself reveal the dynamical aspects of neural communication. This has motivated statistical methods (6–8) for estimating dynamical connectivity from recordings of neurons and brain imaging.

Dynamical connectivity can be inferred indirectly from time-series data and methods for doing so fall into two broad categories, depending on the inference assumptions: functional connectivity (FC) or effective connectivity (EC). EC is based on an underlying generative model of directed connectivity and searches for the best model to account for the data. FC does not assume a model and is based only on statistical analysis of data from brain activity. The distinction between FC and EC is not strict since they both aim to infer the communication pathways in the brain and others have advocated a broader definition of FC to include both (9).

Conventional FC is often evaluated by estimating pairwise covariance, a symmetric measure that cannot detect directional coupling or disambiguate two unconnected nodes confounded with high correlation due to a common input (6, 10). Covariance estimates can be improved by using a suitably regularized partial covariance matrix, which is a global method that can “explain away” some of the ambiguities, but this matrix is symmetric and does not solve the problem of finding the direction of coupling. Another assumption that covariance methods make is that conditions are stationary so that sample covariance can be obtained by averaging across time, but this assumption is violated by changes in the level of brain arousal or tasks when switches occur between internal brain states. Despite these limitations, covariance methods have served as intuitive measures of brain coordination and remain the predominant way that most researchers estimate FC.

More sophisticated causal inference methods have been developed to estimate directed connections (11). In some prevailing methods, statistical causality is based on the degree to which one time series can predict another one. These methods include variants of Granger causality (12), cross-convergent mapping (CCM) (13), and cross-dynamical delay differential analysis (cd-DDA) (14). For example, for Granger causality, time series A is causally related to time series B if the removal of A reduces the accuracy of predicting the future of B. As evident from the definition, selecting a best-fit model for prediction is essential. Other methods, rooted in information theory, such as conditional mutual information (15) and transfer entropy (16, 17), have also been used to infer directed connectivity. This class of methods can in principle improve inference accuracy, but these methods require much more computation or a bigger sample size than covariance methods and do not scale well.

EC models such as dynamic causal modeling (DCM) (8) and Bayes net models (11, 18–20) search the feasible graph space and fit the entire dataset to every hypothesis. Bayes net methods fit multivariate time recordings through static probability distributions without time dependency while DCM models the observations explicitly through the dynamical process that generates the recorded signals and has been the mainstream method to infer EC. In fact, linear dynamic differential covariance (DDC) shares the same assumption as the linearized DCM (7). The graph searching process requires even more computation, severely limiting the size of the network that can be analyzed.

We previously introduced differential covariance (dCov) (21, 22), a directed FC estimation method, and highlighted the performance of two matrices, Δc, which is the correlation between the derivative signal and the signal itself, and Δp, which is the partial covariance between them. In simulated test cases, dCov detected network connections with higher sensitivity than many of the methods reviewed in Smith et al. (6). In this paper, we derive a direct link between dCov and dynamical models of network activity. This leads to a class of estimators called DDC based on an interaction matrix that appears in the equations for a dynamical system. DDC provides a simple and efficient estimate of directed connectivity by combining a model-based approach from EC with computationally efficient methods from FC. DDC is based on an implicit generative model that approximates brain dynamics and entails causality in the context of control theory.

In the following sections, we first analytically show that DDC provides unbiased estimates regardless of the noise structure, without assuming that the data are stationary. These favorable statistical properties were numerically confirmed in networks with both linear and nonlinear dynamics. We then show that DDC can infer the ground-truth connections and their direction in multiscale neural network simulations. The inference accuracy and efficiency were benchmarked against most estimators mentioned above (Table 1). Finally, we apply DDC to resting-state functional magnetic resonance imaging (rs-fMRI) recordings from over 1,000 subjects and show that the extracted connectivity closely matches the structural connectivity measured by diffusion MRI.

**Table 1.** Summary of estimators

Estimator	Notation
Cov	Covariance matrix
P	Partial covariance matrix
L1-reg	L1-regularized partial covariance matrix
L2-reg	L2-regularized partial covariance matrix
Partial-MI	Partial mutual information
c-Granger	Conditional Granger causality
C${}_{spk}$	Spike-train cross-correlogram–based connectivity
Δc	Differential covariance matrix
Δp	Partial differential covariance matrix
ΔL	Linear DDC
ΔR	General nonlinear DDC
ΔReLU	Nonlinear DDC with ReLU nonlinearity

## 2. Results

### A. DDC.

Models of neural dynamics span a wide range of scales. At the microscopic level, the voltage trace, calcium dynamics, and firing rate of a single neuron are highly nonlinear. These dynamics are often modeled using biophysical models based on voltage-gated ion channels. In contrast, at the macroscopic level the collective activity of a population of neurons and interactions between brain regions can be approximated by linear dynamics because of ensemble averaging (7, 23–25). For example, Nozari et al. (25) showed that compared to other sophisticated nonlinear families of models, the simple linear autoregressive model performed best on modeling fMRI and intracranial electroencephalography (iEEG) recordings from hundreds of human subjects.

We first propose a linear dynamical model in Eq. **1** for global recordings and a nonlinear dynamical model in Eq. **2** for local neural recordings:

[1] $$\begin{array}{ll} & \frac{dx}{dt}=Wx \end{array}$$

[2] $$\begin{array}{ll} & \frac{dx}{dt}=WR(x), \end{array}$$

where the column vector **x** is the neural activity, such as the membrane voltage or fMRI signal; **W** is the square connectivity matrix; and R(x) is a nonlinear response function. Taking the outer product of Eqs. **1** and **2** with **x** and time averaging 〈,〉 yields

[3] $$\begin{array}{ll} & \langle \frac{dx}{dt},x\rangle =W\langle x,x\rangle \\ & \Delta \text{L}≔\langle \frac{dx}{dt},x\rangle {\langle x,x\rangle }^{-1} \end{array}$$

[4] $$\begin{array}{ll} & \langle \frac{dx}{dt},x\rangle =W\langle R(x),x\rangle \\ & \Delta \text{R}≔\langle \frac{dx}{dt},x\rangle {\langle R(x),x\rangle }^{-1}, \end{array}$$

where ΔL and ΔR are DDC estimators for **W**. DDC is the least-squares error estimator (LSE) of **W** under the assumed system equations (*Materials and Methods*, *1.G.1*). Potentially, the extensive statistical literature about LSE (26) can be applied to refine DDC estimation. For example, the pseudoinverse could be used if the covariance matrix is rank deficient. More details are provided in *Discussion*.

The origin of the linear DDC estimator ΔL from a dynamical model provides an intuition for its effectiveness in estimating **W** as the product of two matrices: The first term is differential covariance, which carries information about sources and sinks. In a neuron the sink is the inward current from synaptic inputs in the receiving area and in brain imaging it is related to changes in surrogates for local brain activity, whereas the source is the activity level in the sending area. In the second term, an entry in the partial covariance matrix is zero if and only if the residual correlation between $x_{i}$ and $x_{j}$ is zero, which cancels the influence of common sources. Robust estimation of directional interactions becomes possible by combining signals from sources and sinks and canceling signals from common sources. The multiplicative combination of the two terms yields better estimates than either one alone.

A family of estimators arises from the DDC estimator ΔR for nonlinear dynamical systems for different R(x). Estimators can be adapted to the filtering effects from different recording techniques, such as the slow kinetics of calcium signals, by choosing the nonlinear function R(x) appropriately. Here, we use the rectified linear unit (ReLU), parameterized by a threshold, *θ*, that is often used in artificial neural networks (27), yielding ΔReLU as the corresponding nonlinear DDC estimator. The threshold can be set to optimize performance. Intuitively, the ReLU function rectifies low-magnitude “noise” and retains larger signals.

### B. DDC Provides Unbiased Estimation for Nonstationary Data.

**Fig. 1.**
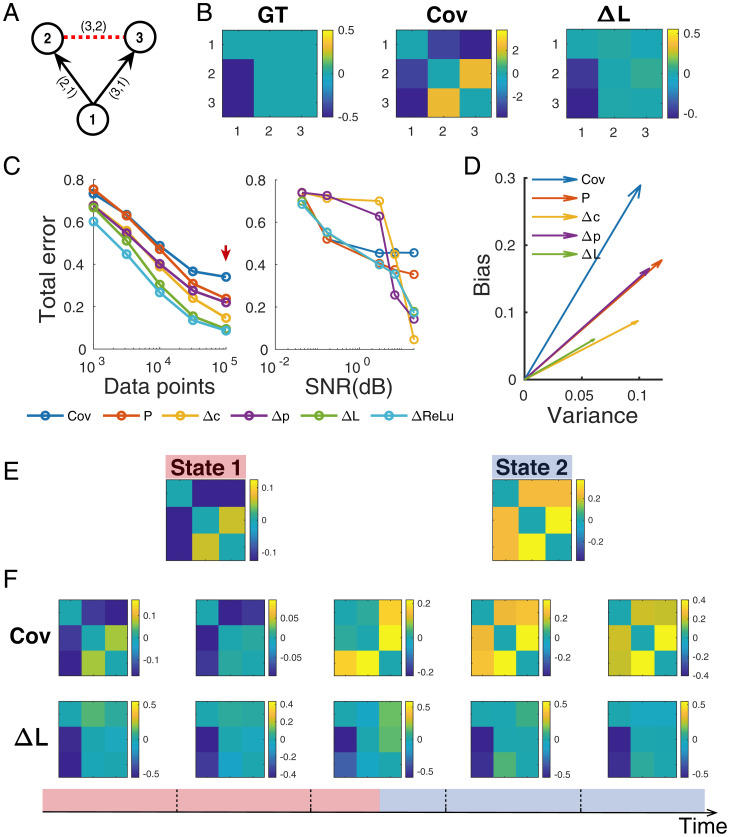
DDC provides unbiased estimation for nonstationary data. (*A*) Ground-truth (GT) network structure. Black solid lines are directed physical connections and red dashed line is false positive connections commonly inferred by covariance estimation. Time series were simulated using linear dynamics. (*B*) GT in matrix form, sample covariance (Cov), and ΔL estimation. (*C*) *Left*: Influence of simulation length on the estimation error, quantified as normalized Euclidean distance between the ground truth and estimation. *Right*: Influence of observational noise, imposed as additive Gaussian random noise. ΔL exhibited low error across all tested data sizes and is robust to noise corruption. (*D*) Orthogonal decomposition of estimation errors into variance and bias computed based on sufficient data from 50 random trials (corresponding to the red arrow in *C*). Bias measures the estimation accuracy while variance measures the precision. ΔL exhibited lower bias, variance, and bias-to-variance ratio. (*E* and *F*) Simulation of the static confounder motif governed by a two-state dynamical system (*Materials and Methods*, *2.A*). (*E*) Analytical solution of the steady-state covariance matrix (*Materials and Methods*, *1.G.3*) in state 1 (*Left*) and state 2 (*Right*). (*F*) *Bottom*: Timeline for sample time series with five nonoverlapping sampling windows. The simulated time series was in state 1 during windows I and II and in state 2 during windows IV and V. Window III included data from both states. Sample covariance estimation (*Top* row) shifted between states; in contrast, ΔL estimation (*Bottom* row) consistently reported the static true connectivity. For a clear illustration, we removed the diagonal values from estimated matrices. Cov, sample covariance estimation; Δc, differential covariance matrix; Δp, partial differential covariance matrix; P, partial covariance estimation; SNR, signal-to-noise ratio.

DDC estimators have several favorable statistical properties. First, given the correct neural model for the generative process, DDC provides an unbiased estimation of the connectivity matrix. In *Material and Methods*, *1.G.2*, we show analytically that in systems governed by stochastic differential equations, DDC gives unbiased estimates of connectivity **W** in the sense that the DDC estimation converges to the ground truth given a sufficient number of trials. In addition, the estimation remains unbiased regardless of the noise structure (**D** in Eq. **13**)—whether or not the added noise is correlated. To numerically confirm this result, we simulated a confounder network governed by linear dynamics (Fig. 1*A*) and estimated the connectivity from simulated time traces (Fig. 1*B*).

It appears that ΔL was able to recover the ground-truth network structure, within a margin of error. To further investigate the origin of error, we orthogonally decomposed the total error (*Materials and Methods*) across trials into bias and variance (Fig. 1*D*). The bias part is due to the intrinsic property of the estimation method while the variance part drops as the number of estimation trials increases. Across 50 simulations, ΔL achieved the smallest estimation error, and more importantly, its bias over variance ratio ($\theta_{b}$) was also the lowest (Fig. 1*D*). Thus, both analytical and numerical results confirmed that DDC estimation is unbiased.

We also quantified the variance and bias over a range of data size and observational noise. DDC consistently had the least estimation error regardless of the size of the dataset (Fig. 1*C*). In contrast, inference bias for covariance (Cov), partial covariance (P), differential covariance (Δc), and partial differential covariance (Δp) diverged as data volume increased, introducing a systematic error. Regarding noise tolerance (Fig. 1*C*), the performance of dCov matrices (Δc and Δp) rapidly deteriorated with increasing noise, probably due to the inaccuracies in the computation of derivative. However, DDC remained robust despite these inaccuracies.

Second, we also prove in *Materials and Methods* that DDC can be used to analyze nonstationary data whose higher-order statistics vary with time. In practical neural data processing, stationarity is often assumed when estimating the covariance matrix through sampling over time. However, this may not be a valid assumption because neural recordings can be quite nonstationary due to fluctuating brain states owing to neuromodulation and varying sensory inputs. There is a need for methods that can analyze nonstationary data. In the derivation of DDC, stationarity was not required because relationships imposed by the system equation hold at every time step, regardless of the probability distribution of the process. To verify that DDC does not depend on stationarity, we simulated a two-state dynamical system (*Materials and Methods*) whose connectivity (shown in Fig. 1*A*) remained time invariant while the noise structure was switched between states. The analytical solution of the covariance structure is given by Eq. **21**, as plotted in Fig. 1*E*. Using nonoverlapping sliding windows, we obtained the sample covariance and ΔL estimation of the connectivity matrix (Fig. 1*F*). The time-varying covariance matrix confirmed that the time trace is not stationary and thus sample covariance estimation fails to capture the true connectivity profile. On the other hand, ΔL consistently and accurately estimated the true connectivity matrix, even in window III (Fig. 1*F*) where state switching occurred.

### C. DDC Inferred the Existence and Direction of Multiple Network Structures and Dynamics.

We applied DDC to a number of dynamical systems, including stochastic nonlinear systems and deterministic chaotic systems. The objective was to show the extent to which the connection strengths and directions can be estimated by DDC in a wide class of dynamical systems, especially by the linear model.

**Fig. 2.**
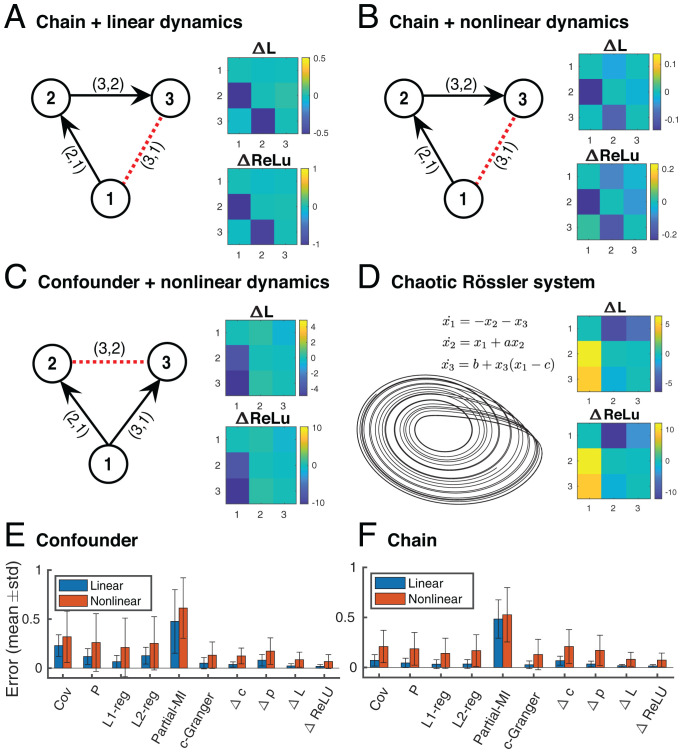
DDC recovers ground-truth connectivity across multiple three-node networks. (*A*–*C*) DDC estimation results based on different network structures (chain and confounder) simulated using linear and nonlinear dynamics. *Left*: Ground-truth network structure. Black solid lines are directed physical connections and red dashed lines are false positive connections commonly inferred by covariance estimation. The edges labeled (i,j), indicating a connection from *j* to *i*, stand for the matrix entry at the *i*th row and *j*th column. *Right*: Estimated ΔL and ΔReLU. (*D*) *Left*: Phase diagram of $x_{1}$ and $x_{2}$ of the Rössler system governed by system equations shown above. *Right*: estimated ΔL and ΔReLU. (*E* and *F*) Estimation error, quantified as normalized Euclidean distance between the ground truth and estimation, over 50 trials for both linear and nonlinear models benchmarked with state-of-the-art network inference algorithms. c-Granger, conditional Granger causality; Cov, sample covariance estimation; Δc, differential covariance matrix; Δp, partial differential covariance matrix; L1/L2-reg, L1/L2-regularized partial covariance matrix; P, partial covariance estimation; partial-MI, partial mutual information; std, SD (standard deviation).

As a proof of principle, we applied DDC to three-node networks with varying dynamics and network structures (Fig. 2). The chain motif (Fig. 2 *A* and *B*) and confounder motif (Fig. 2*C*) were chosen because they both have a node pair (red dashed line) that is highly correlated but with no physical connection, which is an ideal test of whether DDC can “explain away” spurious correlations. We simulated both linear- and sigmoid-based nonlinear dynamics (*Materials and Methods*). We intentionally introduced a model mismatch—“sigmoid”-based generative dynamics and “ReLU” nonlinearity for estimation—because in practice, a perfect match is not possible. Both ΔL and ΔReLU correctly inferred the existence and direction of the ground-truth connections (Fig. 2) while the covariance matrix (Cov) failed to explain away false positive connections and partial covariance (P) was not able to determine the directionality of connections (*SI Appendix*, Fig. S2*B*).

**Fig. 3.**
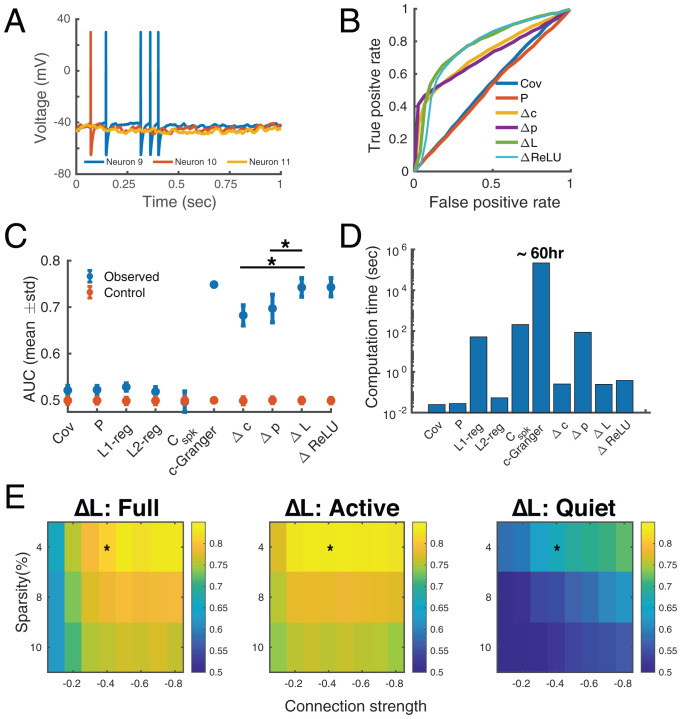
Estimation of performance for spiking networks. (*A*) Selected membrane potential traces simulated using 200 LIF neurons with a sparse Erdös–Rényi random connectivity. (*B*) ROCs quantifying classification performances for true connections (network sparsity, 0.04; connection strength, –0.4; average firing rate, 3.5 Hz). The curves for ΔL and ΔReLU were similar due to the threshold selection process (*Materials and Methods*). (*C*) AUC across 50 realizations of the random graph (*P <0.001, two-sided Wilcoxon rank-sum test). Control values were calculated by generating another realization of the Erdös–Rényi graph with the same sparsity. Only one AUC value was shown for the c-Granger estimate because of the excessive computation needed. (*D*) MATLAB computation time on a computation cluster with 32 CPUs for the dataset of 200 nodes by 200,000 time points. (*E*) AUC values for ΔL applied to networks with a range of sparsities and connection strengths. Estimates used (full) all time points, (active) time points around the spike times, or (quiet) time points outside the spike time intervals. Unsurprisingly, most network communication took place when there were active units. Asterisks indicate the condition used for ΔL in *C*. C**_spk_**: spike-train cross-correlogram–based connectivity.

We further benchmarked DDC performance with more sophisticated methods, including regularized partial covariance (L1-/L2-reg), partial mutual information (MI), and conditional Granger causality (c-Granger) (Fig. 2 *E* and *F*). L1-reg and c-Granger exhibited comparable performance in linear simulations but their performance deteriorated for models with nonlinear dynamics. The heavy computation burden for optimization or model fitting makes these two methods difficult to scale up (see computation time in *SI Appendix*, Fig. S2*E* for a 50-node task and in Fig. 3*D* for a 200-node task). Because regularized partial covariance and c-Granger assume stationarity, they performed poorly on the state-switching case shown in Fig. 1 *E* and *F*.

DDC was also applied to a larger network consisting of 50 nodes and structured by a combination of confounder and chain motifs (*SI Appendix*, Fig. S2*A*). As in the small network case, ΔL and ΔReLU accurately estimated the existence and direction of connections (*SI Appendix*, Fig. S2*B*). L1-reg and c-Granger achieved good performance for models with linear dynamics but performed poorly when the system was nonlinear (*SI Appendix*, Fig. S2*D*). In this 50-node estimation task, c-Granger computation time was more than three orders of magnitude greater than that of the other methods (*SI Appendix*, Fig. S2*E*). Estimation accuracy improved with larger datasets for most of the methods we tested (*SI Appendix*, Fig. S2*C*).

Finally, we asked whether DDC can track the information flow in a nonlinear Rössler system, which has a deterministic chaotic attractor. The three equations for this system in Fig. 2*D* have a nonlinear bidirectional confounder motif. ΔL and ΔReLU correctly identified direct connections and ignored the strong correlations between $x_{2}$ and $x_{3}$. This suggests that DDC estimation is robust to model mismatch and can faithfully reflect the direct interactions in the system equation despite the unpredictability of the chaotic system.

### D. DDC Identified Ground-Truth Connections with High Sensitivity across Multiscale Neural Networks.

Next, we tested DDC on a network model with 200 leaky integrate-and-fire (LIF) neurons (29). These neurons integrate exponentially filtered synaptic inputs until the membrane potential reaches a threshold, which triggers a spike and a reset to resting membrane potential. The connectivity matrix was a globally connected Erdös–Rényi random graph with uniform connection strengths, to test methods for extracting the existence and direction of network edges (*Materials and Methods*). We used the classification performance of true connections with increasing binarization thresholds (*Materials and Methods*) as a surrogate measure for how well the connectivity of the entire matrix was estimated.

Graphs with a range of sparsity and connection strengths were simulated and DDC was applied to the subthreshold membrane potentials (*Materials and Methods*, representative traces shown in Fig. 3*A*). Performance was quantified by the area under the curve (AUC) of specificity versus sensitivity (Fig. 3 *B* and *C*). Directed estimation methods (c-Granger, dCov, and DDC) have higher AUC values because the ground-truth matrices were not symmetric. ΔL and ΔReLU were significantly (*P* <0.001, rank-sum test) better than all other methods except c-Granger, which reached a comparable performance level. However, one trial of c-Granger estimation took ∼60 h on a computing cluster with 32 cores, which makes it impractical to analyze the large-scale neural recordings that have become available. Both the linear and nonlinear estimators presented similar curves (Fig. 3*B*) because the threshold selection process (*Materials and Methods*) of ΔReLU favored a low threshold, approximating a linear model. Possible explanations and improvements of the nonlinear estimator are explored in *Discussion*. Remarkably, the linear DDC estimator retained its high level of performance in this highly nonlinear simulation and was robust to a broad range of network configurations with different sparsity levels and connection strengths (Fig. 3*E*). In general, the additional operation of taking partial covariance compared to pairwise covariance did not improve the performance (compare Cov, Δc with P, Δp in Fig. 3*D*) because in a sparsely connected network structure the influence of indirect connections is weak. Interestingly, Δp had very high sensitivity (true positive rate) even when very few connections were thresholded as positive. This might be due to its sparse estimation (21, 22).

We also tested the methods on simulated recordings of macroscopic neural activities based on the reduced Wong–Wang model of the resting state (30). The interaction matrix involves both self-excitation and experimentally measured long-range structural connectivity. We used two datasets of structural connectome in the simulation: the Human Connectome Project (HCP) diffusion MRI (dMRI) connectome (31) and the diffusion tensor imaging (DTI) dataset built in the virtual brain simulator (TVB) (32). The details of the two datasets and the simulation parameters are summarized in Table 2. Most physiological parameters were taken from Deco et al. (30) unless otherwise noted in Table 2. In the dMRI simulation, the relative connection strength and driving current were slightly tuned to resemble the bifurcation plot of the full spiking network (figure 2 in ref. 30).

**Table 2.** Summary of structural connectivity matrices

Features	DTI	HCP dMRI
Appearance	*SI Appendix*, Fig. S5	Fig. 4
No. of subjects	Population	Population and 1,064 individuals
No. of nodes	76	360
Sparsity, %	3	5
Simulation size	Two hemispheres	One hemisphere
Evaluation size	One hemisphere	One hemisphere
Simulation parameter*	—	*c* = 0.01; $I_{0}=-0.1$

^*^Most parameters are from ref. (30) unless otherwise denoted.

Due to the spike and reset process, the numerical derivative of membrane potential jumps transiently during spike events, thus significantly contributing to DDC estimators. We suspected the DDC estimators performed well because of these spike events. To test this possibility, we split the time trace and its derivative trace into active time points where at least one neuron spikes and quiet time points where no neuron emits a single spike. We extended the simulation to 40 s to ensure a sufficient data volume and quantified DDC performance for both parts (Fig. 3*E*, *Center* and *Right*). Estimation of ΔL using active time points was as accurate as that using full time points. However, the spike train cross-correlogram was itself not enough to recover the connectivity as indicated by the low AUC value of C${}_{spk}$ in Fig. 3*C* and *SI Appendix*, Fig. S3*C*). This points toward the importance of including voltage fluctuations within the active period window, in addition to the binary spike train.

**Fig. 4.**
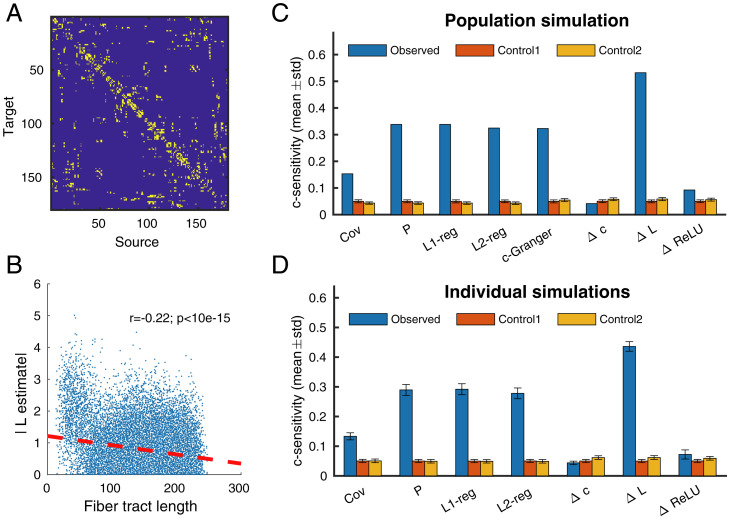
Estimation performance for a macroscopic brain surface model. (*A*) Population-averaged structural connectivity measured by dMRI from one hemisphere. The matrix was threshold into a binary matrix where only the strongest 5% of connections (yellow entries) were kept. (*B*) Correlation between absolute value of ΔL estimate and fiber tract length in the model. Time series were simulated for 1,000 s at 1,000 Hz using a reduced Wong–Wang model supported by population-averaged dMRI connectivity from one hemisphere. (*C*) Estimator performance (observed) quantified through c-sensitivity, a measure of the separation between estimated values of true positive connections and true negative connections. Control 1 was calculated based on 1,000 realizations of an Erdös–Rényi graph with same sparsity. Control 2 was calculated based on 1,000 trials of a degree-preserving randomization algorithm (28). Due to computation limit, “c-Granger” estimation was based on 10% of the simulated data. (*D*) Estimation performance (c-sensitivity) in simulations supported by 1,065 individual dMRI connectivities (one hemisphere).

For the HCP dMRI dataset, we simulated neural dynamics based on both population-level connectivity (Fig. 4*A*) and 1,065 individual connectomes. We thresholded the structural connectivity matrix to a sparsity of 5% because it is difficult to achieve above chance performance for any methods when the connectivity matrix is too dense. Every node in the thresholded graph is still linked to at least five other nodes as shown in *SI Appendix*, Fig. S4*A*. The overall performance was quantified by c-sensitivity (*Materials and Methods*), which is a measure of the separation between the estimated value of true positive connections and the estimated value of true negative connections. (c-sensitivity = 1 when the true positives were completely separated from the others by the estimation.) Chance-level performance was calculated by replacing the ground-truth matrix with either realizations of the Erdös–Rényi graph with the same sparsity level (control 1) or those of degree-preserving randomized graphs (control 2).

In the population-level connectivity simulation, ΔL had the best performance followed by (regularized) precision matrices and c-Granger estimation (Fig. 4*C*), probably because the reduced Wong–Wang model exhibited linear fluctuations around the stable point (30). For c-Granger, only 10% of the data points were used since it took about 46 h to run and full-length estimation would take approximately 1 mo. The ΔL estimated connection strength appeared to be negatively correlated with fiber tract length, in line with most tractography reconstruction observations (31) (Fig. 4*B*). In the 1,065 individual simulations, ΔL continued to exhibit the best performance (Fig. 4*D*).

In another simulation using the built-in structural connectivity in the virtual brain simulator (*SI Appendix*, Fig. S6), ΔL had the highest performance followed by c-Granger and ΔReLU. The c-Granger method had comparable performance but the computation time was almost four orders of magnitude longer, making it impractical. The raw ΔL and ΔReLU matrices uncovered the strongest connections (red arrows) in the ground-truth matrix (*SI Appendix*, Fig. S6*B*). Similarly, only the strongest long-range connections were included, because all methods failed to reach significance for graded anatomical connectivity.

### E. DDC Estimation Is Reliable When Applied to rs-fMRI Recordings.

To critically test DDC on neural data, we applied DDC to rs-fMRI recordings obtained from the HCP. The imaging voxels were parcellated through group independent component analysis (ICA) (*Materials and Methods*), where each independent component (IC) parcellation, shared across subjects, is composed of voxels with similar dynamics. ICs are mainly composed of spatially proximate voxels, forming anatomically recognizable brain regions (*SI Appendix*, Fig. S9). In addition, we focused on the first 46 ICs encompassing over 40% of cortical voxels (*SI Appendix*, Fig. S8*A*) to match cortical measurements using dMRI. Dual regression (*Materials and Methods*) assigned unique ICA-parcellated blood-oxygen-level-dependent (BOLD) signals to each subject, which were treated as nodes for DDC analysis.

**Fig. 5.**
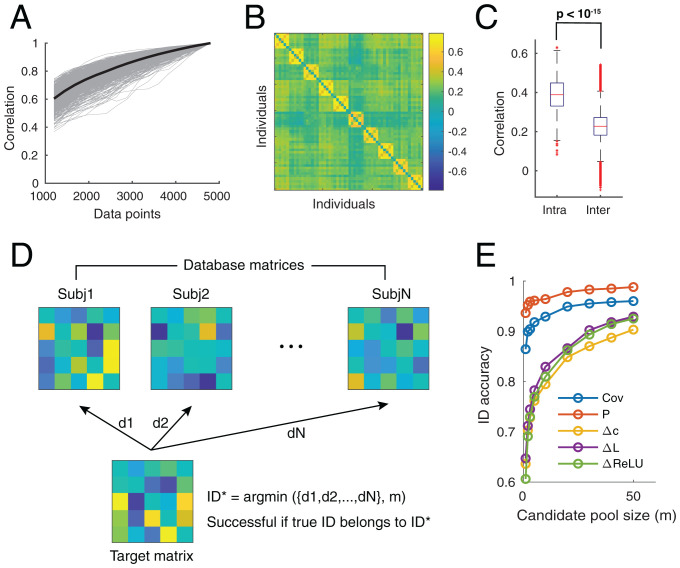
Intra- and interindividual variability of ΔL estimation across scan sessions. (*A*) Correlation of the full-length (4,800 data points) ΔL estimation with that estimated using partial data. One scan session includes 1,200 data points. Each gray curve represents one subject and the black curve is the average. (*B*) Correlation of ΔL estimated using a concatenation of two scan sessions within and between 10 randomly selected subjects. The block diagonal structure indicates a higher level of intraindividual similarity. (*C*) Boxplot of intra- and interindividual correlations pooled from all 1,003 subjects (two-sided Wilcoxon rank-sum test, *P* $<10^{-15}$). The interindividual correlation was calculated based on estimations from nonoverlapping concatenation of sessions [e.g., correlation between ΔL (session 1 and 2) and ΔL (session 3 and 4)]. (*D*) Procedure of subject identification analysis adapted from ref. 33. The subject identity of the target matrix (estimated from two sessions) was inferred based on distance ({d1,d2,…,dN}) between it and database matrices (estimated from the remaining two sessions with known identity). We adopted a soft criterion for identification: The candidate identity pool was constructed as the top *m* subjects with highest correlation. If the true target identity belongs to the candidate pool, then the identification process is successful. ID accuracy was calculated as successful trials over the total number of individuals. (*E*) Identification accuracy, a measure of intraindividual consistency, with respect to candidate pool size (*m*).

We first established the reliability of ΔL estimation, that is, the number of data points required for estimates to become stable and whether the method provided consistent estimates across sessions within one subject. Within one subject, we calculated the correlation of ΔL estimation using all data points with that estimated using different amounts of partial data. The correlation value is 0.6 using data size equivalent to one recording session and gradually increases as more data are included (Fig. 5*A*). Compared to sample covariance estimation (*SI Appendix*, Fig. S7), the data volume requirement is relatively higher probably due to the need to calculate the derivative. For most subjects, 2,400 data points, or equivalently, two sessions, were needed to reach a correlation of 0.8. We further plotted the correlation of ΔL estimation using a concatenation of two sessions within and between 10 randomly selected subjects (Fig. 5*B*). The block structure on the diagonal indicated a higher intraindividual similarity of ΔL across sessions. The population statistics from 1,003 subjects (Fig. 5*C*) confirmed a significantly higher within-subject similarity and a relatively lower between-subject correlation (compare to *SI Appendix*, Fig. S7*C*).

In general, ΔL estimation is more variable than sample covariance estimation. To quantify this, we adapted subject identification analysis (33) (Fig. 5*D*): FC estimation from two sessions together with their subject labels were constructed as database matrices. The identity of a target FC matrix, estimated from the remaining two sessions, was inferred based on the correlation between it and database matrices. To obtain an asymptotic behavior, we adopted a soft criterion for successful identification events. If the true identity belongs to the candidate pool, composed of the top *m* subjects with highest correlation, then the identification process is successful.

As expected, the identification (ID) accuracy increased as the sample pool size (*m*) increased (Fig. 5*E*). Cov and P showed very high ID accuracy as reported in ref. 33. DDC estimators (Δc, ΔL, and ΔReLU) showed an accuracy of around 65% and then reached 90% as pool size increased to 30, which is still acceptable given there are over 1,000 subjects in total. Dynamic FC has been observed previously (34, 35), and identifying its origin might reveal fundamental aspects of brain dynamics.

### F. DDC Consistently Identified Structurally Connected Brain Regions.

**Fig. 6.**
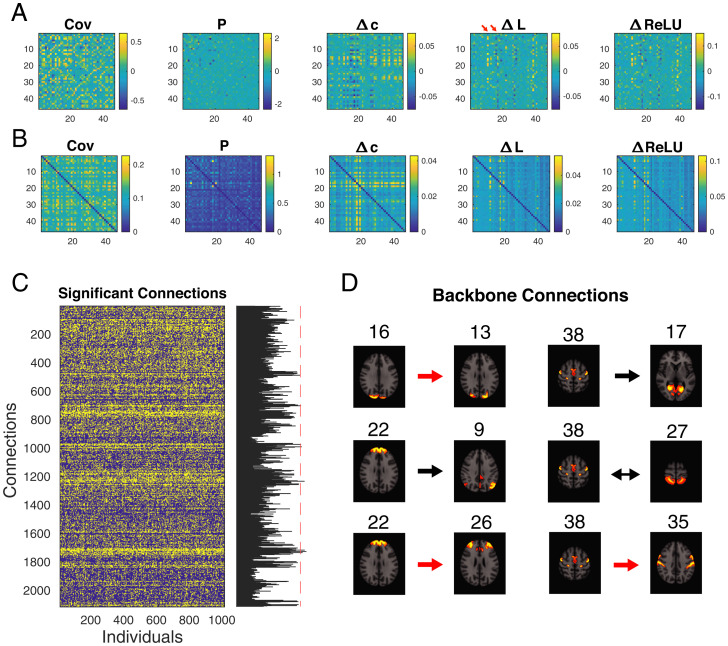
DDC consistently recovered known connections across HCP subjects. (*A* and *B*) Average/SD of estimated FC matrices across HCP subjects. (*C*) Significant (*P* <0.01, yellow entries) ΔL connections for 1,003 individuals. Each column is a binarized FC matrix from one individual reshaped to a column vector. There are important connections shared ubiquitously by the majority of subjects (i.e., “backbone connection”). To systematically select for them, the barplot (*Right*) represents the number of individuals that highlighted a specific connection. The red dashed line represents 90% of total subjects. (*D*) ΔL backbone connection shared by over 90% of subjects and their IC parcellations registered on an MRI template. Arrows indicate the estimated connection direction and the red arrows indicate IC pairs that are anatomically close. Numbers indicate the IC indexes corresponding to *SI Appendix*, Table S1 and Fig. S9.

The average and SD of the estimated interaction matrices across subjects are shown in Fig. 6 *A* and *B*, respectively, where ΔL and ΔReLU were sparser than the covariance matrix. Two nodes (indicated by red arrows) in ΔL appeared to have a broader range of interactions. They were anatomically registered as “occipital pole” and “medial occipitotemporal gyrus,” reflecting the large proportion of visual ICs in the network (*SI Appendix*, Fig. S9 and Table S1). We binarized the estimated matrices based on significance levels to minimize the influence of nonsignificant spurious connections due to random fluctuations in the signal, thereby increasing noise tolerance. The significance test was determined by an autoregressive bootstrapping procedure. The null hypothesis is that each time series was generated by an independent autoregressive process, which was fitted to the observed data. This ensured that the signature power spectra of fMRI recordings were preserved (*Materials and Methods*). The significant ΔL connections (yellow entries, *P* < 0.01) across subjects are shown in Fig. 6*C*. These “backbone connections” shared across a majority of the subjects could be flexibly tuned for network sparsity level. We adopted a strict criterion because we were interested in the most conserved connections shared by over 90% of subjects (red dashed vertical line in Fig. 6*C*). Their IC parcellations were registered on an MRI template (Fig. 6*D*). In this case, backbone connections were identified between ICs from the same anatomical region as well as interregional interactions (marked in red).

**Fig. 7.**
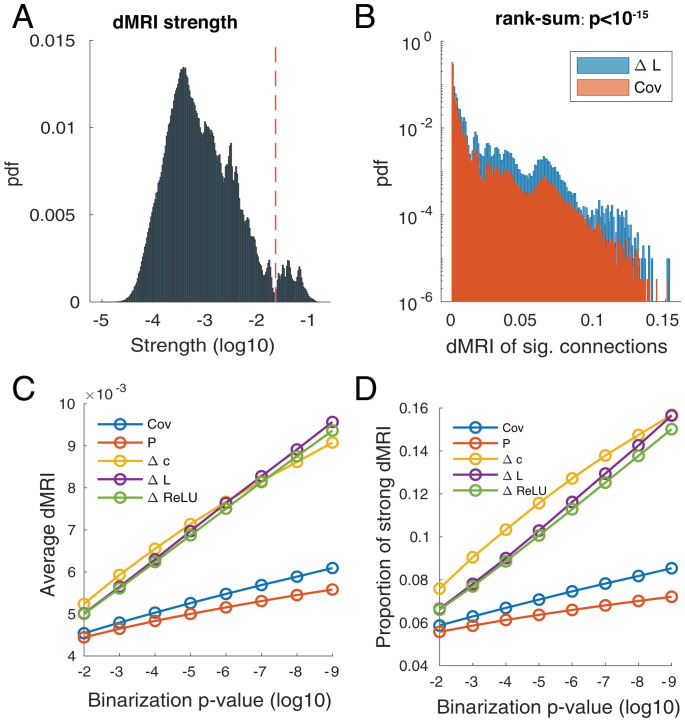
DDC picked up connections with strong dMRI values. (*A*) Distribution of IC-level dMRI strengths. Connections to the right of the cutoff value (red dashed line) were classified as strong connections. (*B*) The dMRI strength distribution of significant ΔL and Cov connections (binarization *P* value $<10^{-9}$). The two distributions are significantly different (two-sided Wilcoxon rank-sum test, *P* $<10^{-15}$). Note the log scale on the *y* axis due to the large abundance of weak connections. (*C*) Average dMRI strength value of significant connections picked by different methods with stricter binarization thresholds. (*D*) Proportion of strong connections. It was used as a supplementary statistic to compare distributions as dMRI strength distribution is almost bimodal.

To quantify the extent to which estimated FCs matched the structural connectivity, we further processed dMRI measurements from the HCP dataset (31) to obtain individual-level IC-based dMRI matrices (*SI Appendix*, Fig. S8 and *Materials and Methods*). At the IC level, dMRI strengths were bimodal (Fig. 7*A*), indicating a clear separation between the strong and weak connections. ΔL identified connections with higher dMRI strength compared to those chosen by the covariance matrix (Fig. 7*B*). Fig. 7*C* shows the increasing average dMRI strength for decreasing binarization threshold, linking the significance of rs-fMRI to dMRI connectivity for all methods and confirming their biological relevance. DDC uncovered connections with significantly higher dMRI strength values than covariance-based methods (Fig. 7*C*) and also identified a larger proportion of strong connections (Fig. 7*D*).

## 3. Discussion

DDC is a promising family of estimators for analyzing the connectivity underlying large-scale brain recordings. Because DDC is derived directly from dynamical system equations that govern neural interactions, no optimization or model fitting is required. DDC is a practical and intuitive method that can be computed rapidly and scales well with the number of recording sites. Unlike methods based on covariance, which are inherently symmetrical, DDC can detect directional interactions and obtain statistical estimates of causality. DDC uncovered ground truth when applied to dynamical simulations of network models and significantly improved estimates of strong dMRI connectivity from rs-fMRI recordings compared with covariance methods. Further analysis is needed to probe the ability of DDC methods to uncover weaker connectivity. Structural anatomy is foundational in neuroscience and functional anatomy that is consistent with the underlying structural anatomy makes possible more accurate predictions about information flow in specific pathways.

The improved performance of DDC on benchmarks had some limitations. First, the parcellation used for analyzing fMRI was based on ICA, which was different from dMRI analysis that used atlas-based parcellation. The additional step to link them through voxel coordinates complicates interpretation of the results. It would have been more parsimonious to use the same parcellation to process both the fMRI and dMRI data as raw voxels, but this would have required reprocessing the raw fMRI or dMRI imaging data. After briefly analyzing another atlas-based fMRI dataset, several nodes were highly colinear with others, leading to a rank-deficient covariance matrix whose inversion made the algorithm unstable. The refactoring of the fMRI data into IC components, as in the HCP dataset and commonly performed in resting-state studies, alleviated the colinearity problem. An alternative approach is simply to discard the dependent nodes as they provide no additional information when perfectly dependent. Algorithms that can invert a rank-deficient matrix could also be applied. For example, the Moore–Penrose pseudoinverse achieves the least-squares estimation of Eq. **3**. However, the inverse of a rank-deficient matrix is not unique, which could result in an infinite number of connectivity estimates. Further constraints such as connection balancing and wiring costs are needed to arrive at a unique estimate.

Another limitation, for both macroscopic brain simulations and HCP fMRI analysis, is that the “ground-truth” matrix (dMRI connectivity) is symmetric due to limitations of diffusion imaging, which precluded harnessing DDC’s capacity to estimate directed connections. Tract tracing, on the other hand, could provide directed structural connections. Two connectome matrices available from macaque brains (figure 2*A* in ref. 36 and figure 11*B* in ref. 37) are also nearly symmetric. Alternatively, directionality can be disambiguated by taking into account known physiological constraints on the directed connections.

The nonlinear version of DDC is underconstrained and more accurate connectivity estimates could be made by adapting the nonlinear function to the specific process generating the neural activity and recordings. For example, a hemodynamic response kernel could be used for fMRI recordings. Here, we adopted a ReLU nonlinearity, which has been widely used to model nonlinear rate-based network models, but its performance on the spiking network model was no better than that on the linear model. A better model for a spiking network would take into account the spiking mechanism and dynamical time constants. (See *Materials and Methods*, *1.G.4* for further discussion on how to modify nonlinear DDC for spiking networks.) In principle, we could also optimize the nonlinear function in a function path space by applying variational calculus. A simpler approach would be to express *R*(*x*) as a linear combination of nonlinear basis functions (such as B-splines) and then optimize the linear coefficients based on model evidence.

Although we focused on the application of DDC to resting-state fMRI, it could also be used to analyze fMRI or EEG time-series data collected during a cognitive task. Brain states during dynamical tasks are often nonstationary. For example, attentional mechanisms and neuromodulatory processes can result in fluctuations of the mean and variance of neural activity. Since DDC does not assume stationarity, it should be robust to these fluctuations and could thus be useful in revealing task-related changes in brain connectivity. This can be implemented by a sliding-window analysis, although temporal precision may be limited as DDC typically requires more data points (on the order of 10^3^ samples) than simpler correlation methods. This limitation can be ameliorated by recording at a high sampling frequency, which is possible for EEG. We also plan to explore the bilinear approximation of dynamical systems in the DCM framework (8). This could reveal not only the endogenous connectivity but also the effects of experimental manipulations on communication between brain regions.

In conclusion, DDC has a number of favorable mathematical properties that should ensure robust estimation of connectivity from a wide range of noisy and nonstationary recordings. Access to the directionality of neural connections opens additional avenues for interpreting the causal flow of information through networks. Identifying connectivity based on dynamical systems models makes direct contact with similar approaches in many other disciplines such as bioengineering, control theory, and network science. DDC should have a broad impact on studies in these areas whenever there is need for estimating directional network connectivity from network activity.

## Materials and Methods

### 1. FC Estimators.

All estimators and abbreviations are summarized in Table 1.

#### A. Covariance-based estimators.

The Cov and P matrices are

[5] Cov=〈x,x〉

[6] $$\text{P}={\text{Cov}}^{-1},$$

where x is a column vector of the system variable and the operation 〈,〉 takes the outer product of two vectors and averages across time. In this paper, all time traces were z scored; thus, the covariance matrix is equivalent to correlation. The covariance matrix reveals only pairwise correlations but the partial covariance matrix controls for confounding effects, one step closer to a causal estimation of global connectivity.

#### B. Regularized partial covariance.

Under the assumption that the connectivity matrix is sparse, regularization methods could be implemented during the regression step of calculating the partial covariance matrix. For example, we calculated L1- and L2-regularized partial covariance matrices through the FMRIB (functional magnetic resonance imaging of the brain) software library (FSL) toolbox (38). To choose the regularization parameter (***λ***), we tested a range of them and chose the one with the best performance with a corresponding quantification metric. We tested λ=5,10,20,50,100,200 for L1 regularization while λ=0.1,0.2,0.5,1,5 for L2 regularization.

#### C. Mutual information.

MI quantified the statistical dependence of two random variables beyond second-order statistics. It is a model-free estimation of dependencies and therefore it should work equally well for both linear and nonlinear simulations. We used the implementation in the Functional Connectivity Toolbox (15) to estimate partial MI since we want to estimate the global network structure.

#### D. Granger causality.

Granger causality (12) defines a statistical interpretation of causality based on predictability: A is said to “Granger cause” B if the predictability of B declines when A is removed from the predictors. The test of predictability increase or decline is usually implemented through multivariate vector autoregressive modeling. We implemented conditional Granger causality through the multivariate Granger causality (MVGC) MATLAB toolbox (39). In this approach, the fundamental assumption is that the time-series data are a realization of a stationary vector autoregressive (VAR) process. The VAR model order was chosen based on the Akaike information criterion and the coefficients of the full/reduced regression model were computed through an ordinary least-squares solution to the regression.

#### E. Spike-train cross-correlograms.

Following Das and Fiete (40) and Guisti et al. (41), we calculated the cross-correlograms based on their Pearson correlation and averaged within a time window (τ=0.1 s) to get a spike-based connectivity measure $C_{spk}$. Specifically, for spike-train $x_{i}$ and $x_{j}$, $g_{ij}(\tau )=\int_{0}^{T-\tau }\bar{x_{i}}(t)\bar{x_{j}}(t+\tau )dt$, where the superscript bar denotes the centered version of the spike train. Then the connectivity value from *j* to *i* was evaluated as

[7] $$C_{\text{spk}}(i,j)=\frac{1}{\tau }\int_{-\tau }^{0}{\text{g}}_{ij}(t)dt.$$

For numerical calculation, we used the sampled time interval (dt = 0.5 ms) to evaluate the integral. For neuron pairs whose firing rates are smaller than 0.1 Hz, their connectivity value was assigned as zero. Around 2,000 connection pairs were skipped in *SI Appendix*, Fig. S3*C*.

#### F. dCov estimators.

Differential covariance (Δc) was calculated as Eq. **8** where $\frac{dx}{dt}$ was numerically computed using a symmetric difference quotient (42). The evaluation of partial differential covariance (Δp) was derived in parallel to partial covariance. The calculation was performed elementwise as in Eq. **9** where Cov refers to the covariance matrix, and *K* denotes the set of all nodes except *i* and *j*:

[8] $$\Delta c=\langle \frac{dx}{dt},x\rangle$$

[9] $$\Delta p_{ij}=\Delta c_{ij}-{\text{Cov}}_{jK}{\text{Cov}}_{KK}^{-1}\Delta c_{iK}^{T}.$$

#### G. DDC.

The definitions of ΔL, ΔR, and ΔReLU can be found in the main text. The parameter θ for **Δ**ReLU was varied from the 5th percentile to the 95th percentile of the z-scored data. The optimal value was chosen based on either the estimation errors (three-neuron simulations) or the AUC values (LIF neuron simulations). In the brain surface model, θ was set to zero.

##### G.1. LSE of the system equations.

The DDC estimators are actually least-squares estimation of the assumed system equations. Let us use the linear system equation as an example:

[10] $$\begin{array}{ll} {\left(\frac{dx}{dt}\right)}_{t} & =Wx_{t}\\ L=\sum_{t}L_{t} & =\sum_{t}{\left[{\left(\frac{dx}{dt}\right)}_{t}-Wx_{t}\right]}^{T}[{\left(\frac{dx}{dt}\right)}_{t}-Wx_{t}] \end{array}$$

To achieve the minimum of square error (*L*), we take the derivative of *L* with respect to W:

[11] $$\begin{array}{ll} \frac{\partial L}{\partial W} & =\sum_{t}\frac{\partial L_{t}}{\partial W}\\ \frac{\partial L_{t}}{\partial W} & =-2{\left(\frac{dx}{dt}\right)}_{t}x_{t}^{T}+2Wx_{t}x_{t}^{T} \end{array}$$

Then setting $\frac{\partial L}{\partial W}=0$, we obtained the LSE of **W**:

[12] $$\begin{array}{ll} \hat{W}\sum_{t}x_{t}x_{t}^{T} & =\sum_{t}{\left(\frac{dx}{dt}\right)}_{t}x_{t}^{T}\\ \Delta \text{L}≔\hat{W} & =\langle \frac{dx}{dt},x\rangle {\langle x,x\rangle }^{-1}. \end{array}$$

In the nonlinear case, the above derivation process holds by replacing $x_{t}$ with $R(x_{t})$. In this view, we could refer to the well-developed statistical theory to improve technical issues such as the singularity of the covariance matrix and the regularization procedure.

##### G.2. DDC derivation for stochastic network models.

To model the randomness in the recorded neural activities, we used stochastic differential equations (SDEs) and evaluated DDC in this stochastic framework:

[13] $$\begin{array}{l} \frac{dx}{dt}=Wx+D\frac{d\beta }{dt}, \end{array}$$

where β is a multidimensional Brownian motion with variance Q and noise structure D influencing the state variable **x**. See Eq. **1** for the definitions of the other terms. The time averages ($\langle x\rangle ≔\sum_{t=0}^{T}x_{t}$) are different from ensemble averages [E(x)] under nonstationary conditions, as analyzed in the next section.

Operating on both sides of this equation with 〈,x〉,

[14] $$\begin{array}{ll} \langle \frac{dx}{dt},x\rangle & =W\langle x,x\rangle +D\langle \frac{d\beta }{dt},x\rangle \\ E\langle \frac{dx}{dt},x\rangle & =WE\langle x,x\rangle +DE\langle \frac{d\beta }{dt},x\rangle . \end{array}$$

To evaluate $\langle \frac{d\beta }{dt},x\rangle$, we first write down the explicit solution of the linear SDE starting at t=0 and then time average both sides:

[15] $$\begin{array}{ll} x_{t} & =\exp\;(Wt)x_{0}+\int_{0}^{t}\exp\;(W(t-\tau ))Dd\beta_{\tau }\\ \langle \frac{d\beta }{dt},x\rangle & =\langle \frac{d\beta }{dt},\exp\;(Wt)x_{0}\rangle +\langle \frac{d\beta }{dt},\int_{0}^{t}\exp\;(W(t-\tau ))Dd\beta_{\tau }\rangle \\ & =W_{T}+B_{T} \end{array}$$

The first term $W_{T}$ is the summation of time-dependent linear Brownian increments, and thus the mean is zero and the variance is a time-dependent scaling of the Brownian variance:

[16] $$\begin{array}{ll} E(W_{T}) & =0\\ Var(W_{T}) & =Q\langle \exp\;(Wt)x_{0},\exp\;(Wt)x_{0}\rangle . \end{array}$$

The second term $B_{T}$ was evaluated using the Ito integral. Because Brownian motion is nowhere differentiable on its path, we numerically approximated the time derivative that we used in the simulations. If we assume ${\left\{t_{k}\right\}}_{k=1}^{\infty }$ as a partition of [0,t] whose partition size is infinitesimal as n→∞, we can compute the Ito integral in the limit. For simplicity, define $\Phi_{\tau }=\exp\;(W(t-\tau ))D$. Then the two terms are composed of nonoverlapping Brownian increments

[17] $$\begin{array}{ll} {\left(\frac{d\beta }{dt}\right)}_{t} & =\frac{\beta_{t+dt}-\beta_{t}}{dt}\\ \int_{0}^{t}\Phi_{\tau }d\beta_{\tau } & =\underset{n\to \infty }{\lim\;}\sum_{k}\Phi (t_{k})[\beta_{t_{k+1}}-\beta_{t_{k}}] \end{array}$$

from which it follows that $E(B_{t})=0$ because Brownian motion has independent and stationary increments:

[18] $$\begin{array}{ll} B_{T} & \propto \sum_{t}\underset{n\to \infty }{\lim\;}\sum_{k}\left(\beta_{t+dt}-\beta_{t})[\Phi (t_{k})(\beta_{t_{k+1}}-\beta_{t_{k}}\right){]}^{T}\\ E(B_{T}) & =0. \end{array}$$

Taken together, the first-order statistics of our linear DDC estimator become

[19] $$\begin{array}{ll} E\langle \frac{dx}{dt},x\rangle & =WE\langle x,x\rangle \\ E(\Delta L) & =E(\langle \frac{dx}{dt},x\rangle {\langle x,x\rangle }^{-1})=W. \end{array}$$

This derivation confirms that DDC is unbiased in the presence of noise as a linear combination of Brownian motion. Simulations of the linear three-neuron model revealed that $\langle \frac{d\beta }{dt},x\rangle$ is at least 10 times smaller in magnitude than 〈x,x〉 even for very high noise variance Q. Remarkably, DDC can still recover the ground-truth connectivity for correlated noise structure (D in Eq. **13**).

##### G.3. Nonstationary conditions.

A continuous-time stochastic solution of the SDE, ${\left\{x_{t}\right\}}_{t=0}^{T}$, is stationary when its finite-dimensional joint distribution is time invariant, which implies that its mean and covariance remain constant across time. Only under the stationary assumption can the covariance matrix then be estimated by the time-averaged sample covariance.

The above SDE framework allows the mean and covariance of state variables to vary with time according to the Ito formula

[20] $$\begin{array}{ll} \frac{dm}{dt} & =Wm\\ \frac{dP}{dt} & =WP+PW^{T}+DQD^{T}, \end{array}$$

where m=E(x), P=Var(x). The process is stationary if the right-hand sides are zero. The steady-state solution of m is either zero or within the null space of W. Meanwhile, the steady-state solution of W is given by the Lyapunov matrix equation and can be solved by vectorization (${}_{v}$) and Kronecker product (⊗):

[21] $$P_{v}=-{\left(I⊗A+A⊗I\right)}^{-1}(D⊗D)Q_{v}.$$

Under nonstationary conditions, 〈x,x〉 is no longer a valid estimate of the covariance matrix. Because the system equation holds at every time step regardless of stationarity, our DDC estimators remain valid and unbiased. These properties make DDC a robust and efficient estimator of FC.

##### G.4. Modifications of the nonlinear estimator.

Models of spiking neurons have several features that are not included in the models studied in this paper. First, decay time constants for neurons and synapses are an important part of their dynamics, such as the leaky dynamics of LIF models. Consider the following linear and nonlinear system equations that include membrane time constants for temporal filtering:

[22] $$\begin{array}{ll} \tau \frac{dx}{dt} & =-x+Wx\\ \tau \frac{dx}{dt} & =-x+WR(x) \end{array}$$

For the linear case, DDC estimation differs only up to a scaling factor and the diagonal terms:

[23] $$\Delta \text{L}=\langle \frac{dx}{dt},x\rangle {\langle x,x\rangle }^{-1}=\frac{1}{\tau }(W-I).$$

For the nonlinear system, the time decay introduces a new term:

[24] $$\begin{array}{ll} \tau \langle \frac{dx}{dt},x\rangle & =-\langle x,x\rangle +W\langle R(x),x\rangle \end{array}$$

[25] $$\Delta \text{D}=(\tau \langle \frac{dx}{dt},x\rangle +\langle x,x\rangle ){\langle R(x),x\rangle }^{-1},$$

where ΔD is a different DDC estimator of W for the temporally filtered nonlinear system. It is proportional to a weighted average of dCov and Cov. The time constant in Eq. **25** is a free parameter that can be estimated or optimized. This approach allows for a better match with the circuit mechanisms that generate the activity and how that activity is transformed by the recording techniques.

A second modification to DDC concerns the sparsity of the spiking. For the simulated LIF network, the sparse spike trains were filtered with decaying synaptic currents. As a consequence, 〈R(x),x〉 was rank deficient and thus noninvertible. One potential remedy is to find the least-squares solution of a sequence of linear equations to optimize W. This will be explored in a future study.

### 2. Simulations of Neural Systems.

#### A. Neural motif dynamics.

We tested the performance of these methods in networks structured to have typical false positive motifs—chain (Fig. 3 *A* and *B*) and confounder (Fig. 3*C*)—with different dynamics and in another Rössler chaotic system. To stabilize the simulation, all nodes had decaying dynamics and they were linked by inhibitory connections. Specifically, the diagonal entries in the ground-truth matrix were set to –1 and connection strength was set to –0.5. We tested connection strength from –0.1 to –1 and it did not affect the estimation results qualitatively.

For linear dynamics, system variable x was simulated through Euler integration according to Eq. **26**, where u is the Gaussian-distributed random drive and ϵ is the Gaussian-distributed observational noise, both independent from x. The integration step is 0.01 s and the length of simulation is 1,000 s unless otherwise specified:

[26] $$\begin{array}{l} \begin{array}{ll} \frac{dx}{dt} & =Wx+u,\;u∼N(0,\sigma^{2})\\ x_{\text{obs}} & =x+\epsilon ,\;\epsilon ∼N(0,\sigma_{\text{obs}}^{2}). \end{array} \end{array}$$

For nonlinear dynamics, simulation was governed by Eq. **27**, where we used a centered sigmoid function to simulate the nonlinearity. The sigmoid function was shifted to have mean of zero because otherwise the inhibitory signal would be too strong in the network and the signal would decay to zero in a short time interval. In the expression of R(x), slope α controls the level of nonlinearity in the network and was set to 1 by default. Note the mismatch between simulation nonlinearity and the estimation nonlinearity. The integration step is 0.1 ms and signals were down-sampled to 100 Hz after estimation. Simulation length is 1,000 s unless otherwise mentioned:

[27] $$\begin{array}{ll} \frac{dx}{dt} & =WR(x)+u,\;u∼N(0,\sigma^{2})\\ R(x) & =\frac{1}{1+e^{-\alpha x}}-\frac{1}{2}. \end{array}$$

The equations for the Rössler system are

[28] $$\begin{array}{ll} \frac{dx_{1}}{dt} & =-x_{2}-x_{3}\\ \frac{dx_{2}}{dt} & =x_{1}+ax_{2}\\ \frac{dx_{3}}{dt} & =b+x_{3}(x_{1}-c), \end{array}$$

where $x={\left[x_{1},x_{2},x_{3}\right]}^{T}$, a=b=0.2, and c=5.7. This set of parameters was originally used by Rössler to study the behavior of its chaotic dynamics. The signal was integrated at the step of 0.01 s for 1,000 s. The first 100 s of transient dynamics were discarded.

To simulate a nonstationary system with time-invariant connectivity pattern, we borrowed the idea of hidden Markov models and simulated a two-state dynamical system. The system was simulated for 1,000 s using Euler integration governed by Eq. **29**. Up until 500 s, the noise structure remained to be the identity matrix. After 500 s, we switched the noise structure to $D_{2}$. The steady-state covariance structure of these two states could be analytically evaluated using Eq. **21** and is plotted in Fig. 2*A*:

[29] $$\begin{array}{ll} \frac{dx}{dt} & =Wx+Du,\;u∼N(0,\sigma^{2})\\ D_{1} & =I,\;D_{2}=\left[\begin{array}{ccc} 1 & 0 & 1\\ 1 & 1 & 0\\ 0 & 1 & 1 \end{array}\right]. \end{array}$$

#### B. Sparse LIF network.

The connectivity matrix was constructed as an Erdös–Rényi random graph: Two nodes being connected has probability equal to network sparsity. All connected edges were assigned to have the same strength. Thus, the connectivity matrices were parameterized by only sparsity level and connection strength. LIF neurons could be described by Eq. **30** with double-exponential filtered synapses (29). Once membrane voltage *V* reaches a threshold $V_{\mathrm{thres}}$, the neuron will emit a spike and reset the membrane potential to $V_{\mathrm{reset}}$. The spike train was described by $\sum_{t_{k}<t}\delta (t-t_{k})$ and then filtered to generate synaptic current $r_{i}$. We used subthreshold membrane potential as the system variable (x) of interest. We simulated networks with 200 neurons. The integration process was performed at the step of 0.05 ms, down-sampled to 2,000 Hz, and simulated for 20 s unless otherwise mentioned:

[30] $$\begin{array}{ll} \tau \frac{dV}{dt} & =-V+Wr+I_{\text{BIAS}}\\ \frac{dr_{i}}{dt} & =-\frac{1}{\tau_{d}}r_{i}+h_{i}\\ \frac{dh_{i}}{dt} & =-\frac{1}{\tau_{r}}h_{i}+\frac{1}{\tau_{d}\tau_{r}}\sum_{t_{k}<t}\delta (t-t_{k}) \end{array}$$

#### C. Anatomically supported brain surface model.

Regional brain activities were simulated using a reduced Wong–Wang model (30) (Eq. **31**), where state variable $S_{i}$ denotes the dynamics of the synaptic gating variable and $H(x_{i})$ is the population firing rate. This model is a dynamic mean-field approximation of numerous local spiking units and was applied to efficiently model resting-state dynamics. It involves both self-excitation and long-range experimentally measured connections. The state variable was simulated at 1,000 Hz and the simulation length was 100 s:

[31] $$\begin{array}{ll} \tau \frac{dS_{i}}{dt} & =-\frac{S_{i}}{\tau }+(1-S_{i})\gamma H(x_{i})+\sigma \nu_{i}\\ H(x_{i}) & =\frac{ax_{i}-b}{1-\exp\;(-d(ax_{i}-b))}\\ x_{i} & =cJS_{i}+GJ\sum_{j}W_{ij}S_{j}+I_{0} \end{array}.$$

### 3. Estimator Performance Quantification.

#### A. Variance and bias.

Following Das and Fiete (40), we decomposed the estimation error into variance and bias (*SI Appendix*, Fig. S1). In most cases, the estimation is different from the ground-truth matrix by a scale. So we normalized both estimated and ground-truth matrices between –1 and 1. In addition, dCov-based estimators are directed estimators while covariance-based ones are not. For fair comparison, we considered only the estimation of the lower triangle part where all ground-truth connections are located.

After scaling and lower triangle restriction, estimation error, variance, and bias were calculated as Eq. **32**, where W, $\hat{W}$, and $\bar{W}$ are ground-truth matrix, estimated matrix, and the average of estimated matrices across trials and ||.|| is the vector L2 norm. It is easy to verify that $E\mathrm{rro}r^{2}=Bias^{2}+V\mathrm{arianc}e^{2}$ and thus the vector forms of bias and variance are orthogonal to each other. We measure the relative contribution of bias by the angle ($\theta_{b}$, Eq. **33**) between the vectors associated with bias and variance. Fifty repetitive trials were used across all simulations:

[32] $$\begin{array}{ll} \text{Error} & =\frac{\left||W-\hat{W}|\right|}{\left||W|\right|}\\ \text{Bias} & =\frac{\left||W-\bar{W}|\right|}{\left||W|\right|}\\ \text{Variance} & =\frac{\left||\hat{W}-\bar{W}|\right|}{\left||W|\right|} \end{array}$$

[33] $$\theta_{b}=\tan^{-1}(\frac{\text{Bias}}{\text{Variance}}).$$

#### B. Sensitivity and specificity.

To evaluate the estimation performance in LIF networks, connection recovery sensitivity and specificity were calculated since the networks have sparse connection and the connection strengths are uniform. To be more specific, the estimated matrices were binarized based on their absolute values to determine the existence of connections, which were then compared with the ground-truth connections. We used the absolute value because we cared only about the presence of a connection. Sensitivity and specificity were calculated as the true positive rate and one minus the false positive rate. Varying the binarization threshold gave rise to the receiver operator curve (ROC). The area under the ROC, calculated by trapezoidal integration, indicates the method’s general performance in classifying connections.

For performance evaluation in the brain surface model, c-sensitivity (6) (equation 17 in ref. 43) was adopted. It is defined as the fraction of the estimated true positive values that are higher than the 95th percentile of the false positive values. Like ROC, c-sensitivity quantitatively estimated how sensitive methods are to estimating the presence of a connection. Thus, the absolute values of the estimated matrices were used here.

### 4. HCP Dataset.

#### A. Extracting time traces from rs-fMRI recordings.

We used the extensively processed “HCP1200 Parcellation + Timeseries + Netmats (1003 Subjects)” dataset available through the website (https://www.humanconnectome.org). Detailed preprocessing and study design could be easily accessed through the website. In this release, 1,003 healthy adult human subjects (ages 22 to 37 y, 534 females) were scanned on a 3-T Siemens connectome-Skyra scanner (customized to achieve 100 mTm**^–1^** gradient strength). Each subject underwent 4×15 min recording sessions with temporal resolution of 0.73 s and spatial resolution of 2 mm isotropic.

For imaging data processing, each 15-min run of each subject’s rs-fMRI data was preprocessed according to Smith et al. (44); it was minimally preprocessed (45) and had artifacts removed using ICA and FMRIB’s ICA-based X-noiseifier (FIX) (46, 47). Intersubject registration of cerebral cortex was carried out using areal-feature–based alignment and the multimodal surface matching algorithm (“MSMAll”) (48, 49). Each dataset was temporally demeaned and had variance normalization and then was fed into the MIGP algorithm, whose output is the top 4,500 weighted spatial eigenvectors from a group-averaged principal component analysis (PCA) (a very close approximation to concatenating all subjects’ time series and then applying PCA) (50). The MIGP output was fed into group ICA using FSL’s MELODIC tool, applying it at several different dimensionalities (D = 25, 50, 100, 200, 300). In our analysis, we used the 100-dimension decomposition.

For a given parcellation (group-ICA map), the ICA spatial maps were used to derive one representative time series per IC per subject. This process was fulfilled by the standard “dual-regression stage-1” approach, in which the full set of ICA maps was used as spatial regressors against the full data (51). This results in an N×T (number of components × number of time points) matrix for each subject. Thus, we consider each IC as a network node.

#### B. Intra- and interindividual variability.

The intraindividual variability was quantified as the correlation between DDC estimations across scans. Since DDC requires data points from at least two scans to achieve a relatively high consistency, we concatenated data from two scans for DDC estimation and compared them with the estimation results from the other two remaining scans (Fig. 5*C*). For interindividual variability, estimation results of all six different combinations of concatenation were compared.

#### C. Significance test of the estimated connections.

To assess the statistical significance of the estimated connection, we used an autoregressive (AR) bootstrap procedure (52, 53) to preserve the power spectrum density of BOLD signals. For a specific estimated connection, denoted as element (i,j), our null hypothesis was that signals $x_{i}$ and $x_{j}$ are independent regardless of other nodes’ influence. To generate null time series, we fitted separate AR processes of model order *q* to node-specific time traces. The model order *q* was determined according to the Bayesian information criterion (BIC). A higher-order model was rejected if it could not decrease BIC by more than 2. Using the estimated AR coefficients of empirical time series, we generated 1,000 surrogate null time series and then computed the associated FC corresponding to the null hypothesis. For each connection, we assumed a Gaussian distribution of the null connectivity values generated from null time traces. The *P* value was calculated as the probability of the empirical value that appeared under the null Gaussian distribution. In this paper, we adopted a sequence of significance levels to binarize the matrix so that we could investigate the network behavior asymptotically.

#### D. Individual-level dMRI strength.

To compare the FC metrics to the underlying corticocortical white matter connectivity, we reorganized our previously published diffusion-MRI–based structural connectome (31) in which connectivity was assessed among the 360 cortical areas of the HCP multi-modal parcellation version 1.0 (MMP1.0) atlas (49). Of the 100 IC nodes, 46 are composed of at least 40% cortical voxels (*SI Appendix*, Fig. S8*A*) and as the dMRI connectome was restricted to corticocortical relationships, we limited the scope of our analyses to these nodes. Because the IC nodes have a greater spatial extent than the atlas areas, each is composed of several areas, in whole or in part (mean = 28.3 areas). For each IC node pair, dMRI connectivity was assessed by obtaining the average of the nodes’ constituent interareal connectivity weighted by the fraction of the node pair’s voxels assigned to each areal pair. In cases where an atlas area was partially present in both IC nodes of a pair, that area was excluded from the mean as short-range intra-areal anatomical connectivity was not available.

## Supplementary Material

Supplementary File

## Data Availability

See *SI Appendix*, Table S1 and Figs. S1–S9 for supporting information. Implementation of DDC, network simulation, and HCP processing scripts are all available through GitHub (https://github.com/yschen13/DDC). Previously published data were used for this work (Human Connectome Project).
