# Supplementary material for: Dynamical differential covariance recovers directional network structure in multiscale neural systems
Source: Proc Natl Acad Sci U S A. 2022 Jun 9;119(24):e2117234119. doi: 10.1073/pnas.2117234119 (PMC9214501; doi:10.1073/pnas.2117234119)
Supplement: Supplementary File [file pnas.2117234119.sapp.pdf]

PNAS
www.pnas.org

Supplementary Information for

Dynamical differential covariance recovers directional network structure in multiscale neural systems

Yusi Chen, Burke Q. Rosen and Terrence J. Sejnowski

Yusi Chen (cyusi@ucsd.edu); Terrence Sejnowski (terry@salk.edu)

This PDF file includes:

Figs. S1 to S9

Table S1

Table S1. Anatomic annotations of the 46 ICs. First column is the index number of ICs. Second column is the manually registered anatomical region. DMN: default mode network; NA: no reasonable region identified

Component number	Anatomical Region	Sub-network
1	Occipital Pole	Visual Network
2	Inferior Parietal Lobe	DMN
3	Lateral Occipital Cortex	Visual Network
4	Cuneal Cortex/Occipital Pole	Visual Network
5	Supramarginal Gyrus	DMN
6	Lateral Occipital Cortex	Visual Network
7	Supramarginal Gyrus	DMN
8	Lateral Occipital Cortex	Visual Network
9	Inferior Parietal Lobe	DMN
10	Medial Prefrontal Cortex/Anterior Cingulate Cortex/Lateral Temporal Cortex	DMN
11	Lingual Gyrus/medial occipitotemporal gyrus	Visual Network
12	Angular Gyrus	DMN
13	Occipital Pole	Visual Network
14	Lateral Occipital Cortex - Left	Visual Network
15	Precuneous Cortex	Other Network
16	Occipital Pole	Visual Network
17	Lingual Gyrus/medial occipitotemporal gyrus	Visual Network
18	Lateral Occipital Cortex	Visual Network
19	Occipital Pole	Visual Network
20	Inferior Parietal Lobe	DMN
21	Precentral Gyrus	sensorimotor network
22	Orbital Frontal Cortex	DMN
23	Postcentral Gyrus	sensorimotor network
24	Lateral Occipital Cortex - Right	Visual Network
25	Occipital Pole - Left	Visual Network
26	Frontal Pole	Attention Network
27	Superior Parietal Lobule	Attention Network
28	Hippocampus/Parahippocampal Cortex	DMN
29	Lateral Temporal Cortex	DMN
30	Lateral Occipital Cortex	Visual Network
31	Orbital Frontal Cortex/Lateral Temporal Cortex	Other Network
32	Occipital Pole	Visual Network
33	Inferior Parietal Lobe – Left	DMN
34	Lateral Occipital Cortex	Visual Network
35	Postcentral Gyrus	sensorimotor network
36	Medial Prefrontal Cortex/Anterior Cingulate Cortex	DMN
37	Orbital Frontal Cortex/Lateral Temporal Cortex	DMN
38	Precentral Gyrus / Juxtapositional Lobule Cortex (formerly Supplementary Motor Cortex)	sensorimotor network
39	Occipital Pole	Visual Network
40	Middle Frontal Gyrus	Attention Network
41	Postcentral Gyrus - Left	sensorimotor network
43	Postcentral Gyrus - Right	sensorimotor network
44	Orbital Frontal Cortex	DMN
45	Superior Temporal Gyrus	sensorimotor network
47	NA	NA
48	Frontal Pole	Attention Network

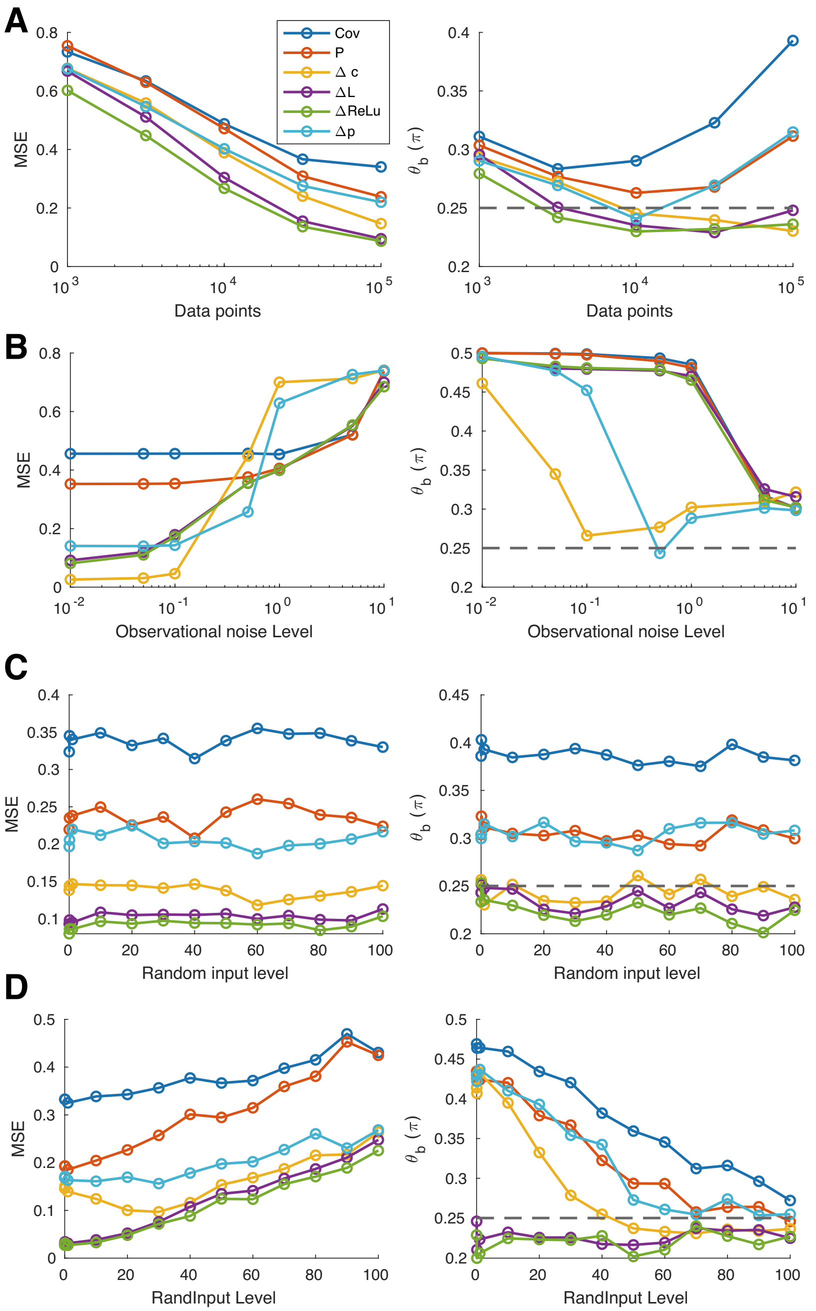

Fig. S1. Estimator variance and bias under different parameter configurations (A) Left: Influence of data volume calculated across 50 trials simulated using linear dynamics and the confounder motif; Right: Contribution of estimation bias (θ_b). $\theta_b = 0.25\pi$ refers to equal bias and variance. In general estimation errors decreased with data volume and covariance based estimators are more biased. (B) Influence of observational noise (horizontal axis is σ_{obs} in Eq. 23) across 50 trials simulated using linear dynamics and the confounder motif. Δc and Δp performance deteriorated with larger amount of noise while ΔL and ΔReLU remained robust under the influence of observational noise. (C) Influence of random input strength (horizontal axis is σ in Eq. 23) across 50 trials simulated using linear dynamics and the confounder motif. Input strength had limited effect on estimator performance since DDC is an unbiased estimator. (D) Influence of random input strength across 50 trials simulated using highly nonlinear dynamics (α=50 in Eq. 24) and the confounder motif. In general, the higher the input strength, the worse the estimation performance. Note the superior performance of ΔL and ΔReLU over all other estimators when the random input strength was relatively low.

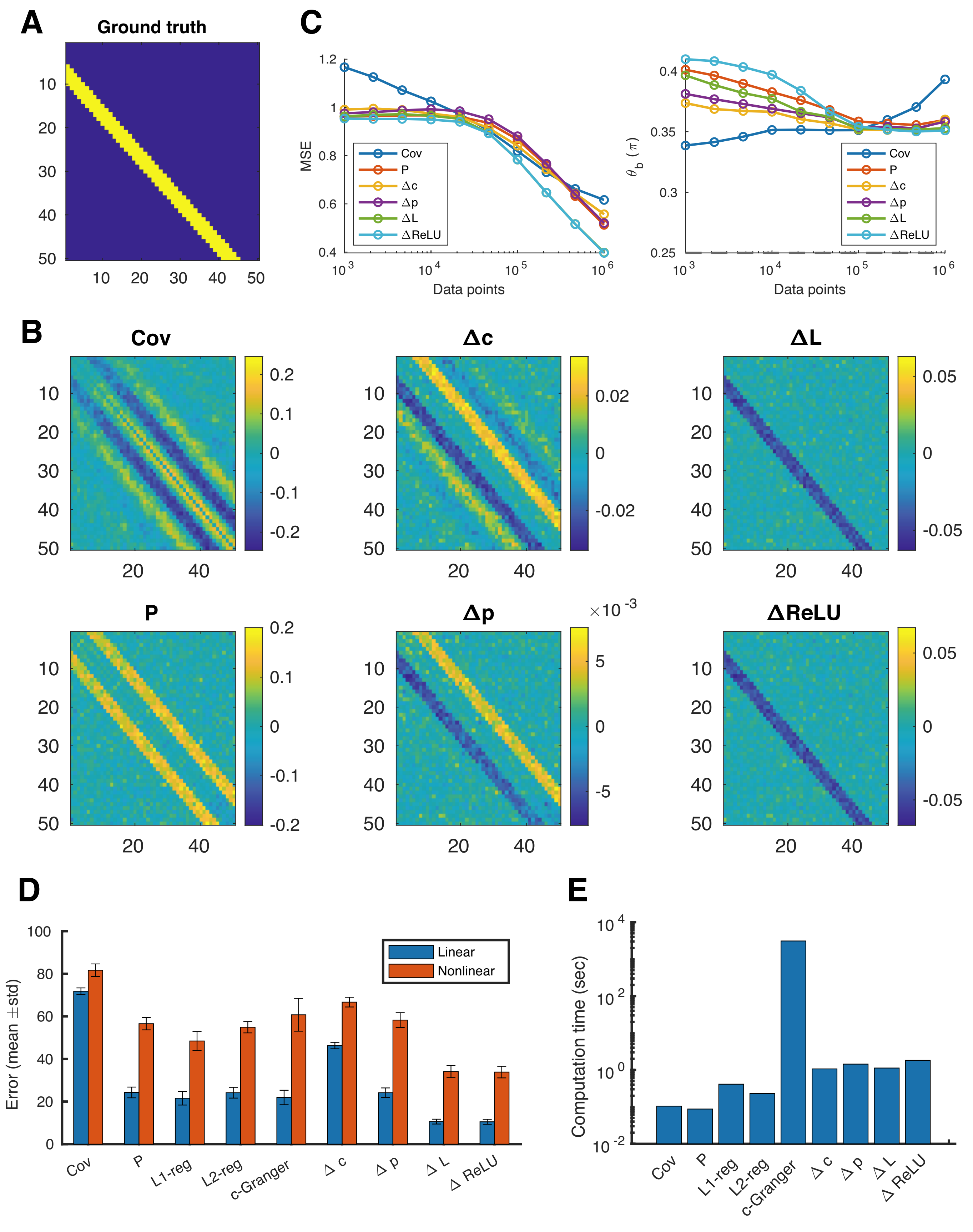

Fig. S2. Estimator performance scaled up to larger networks. (A) Ground truth connectivity pattern of a 50-node network. The connected edges were shown as yellow entries and they were assigned negative strength to stabilize simulation. (B) Estimated matrices using a sufficiently large data volume (10^6 data points) and nonlinear dynamics (Eq. 24), which is the harder scenario according to Fig. S1. ΔL and ΔReLU had cleaner estimation of the ground truth matrix. (C) Influence of dataset size on performance. (D) Estimation performance of both linear and nonlinear simulation. (E) Computation time in seconds.

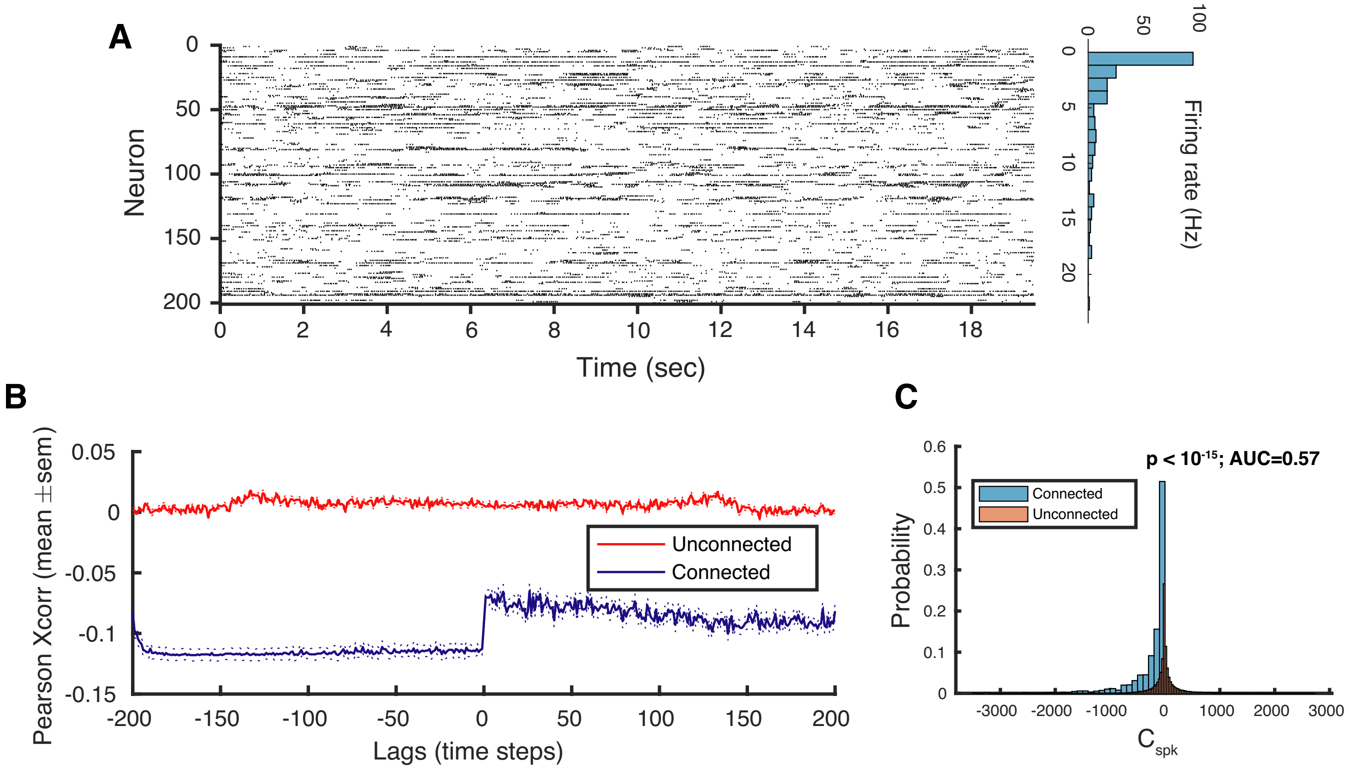

Fig. S3. Connectivity estimation using spike trains (A) Left: The spike train of 200 simulated neurons for 20 seconds (network sparsity: 0.04, connection strength: -0.4). Right: Histogram of the firing rate. (Average: 3.5Hz, Quiet neurons: 29). (B) Pearson cross correlogram of connected pairs and unconnected pairs. C_{spk} was calculated by averaging the cross correlation for the negative time lags. (C) Connected pairs have significantly (rank-sum $p < 10^{-15}$) smaller C_{spk} values compared to unconnected pairs. But the AUC (=0.57) value remained to be low.

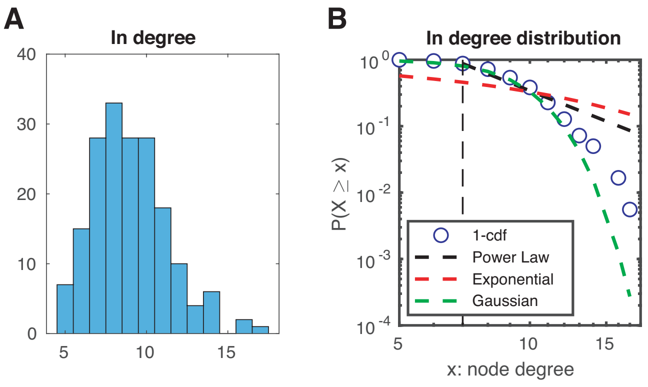

Fig. S4. (A) Incoming degree distribution of the thresholded connectivity matrix (Fig.4A); (B) Average neural activity level across time.

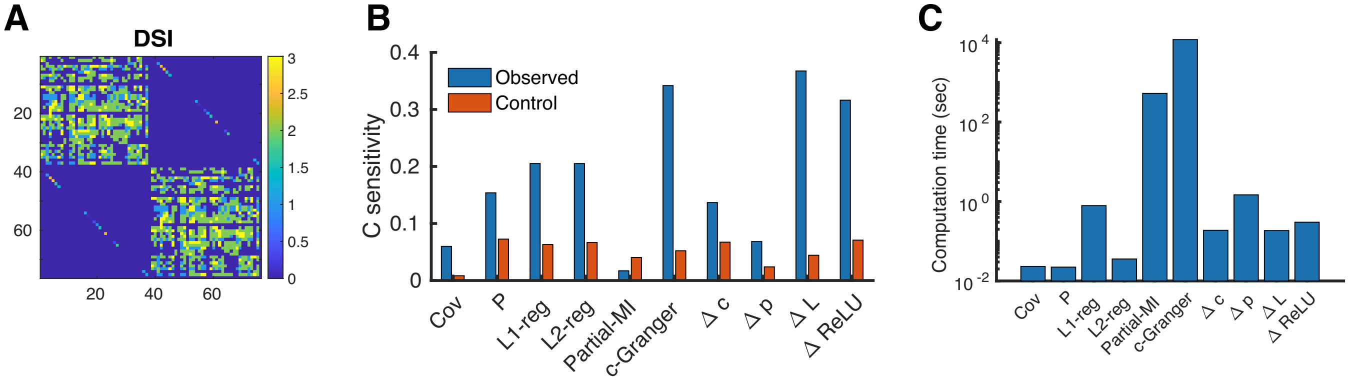

Fig. S5. Estimation performance for a macroscopic brain surface model. (A) Long-range structural connectivity measured by Diffusion Spectrum Imaging (DSI). The block structure represents the left and the right hemispheres. (B) C-sensitivity of the estimators, a measure of the separation between estimated values of true positive connections and true negative connections. Control values were calculated by randomly permuting the edges in the ground truth network. (C) Computation time for the dataset of 76 nodes by 100,000 time points.

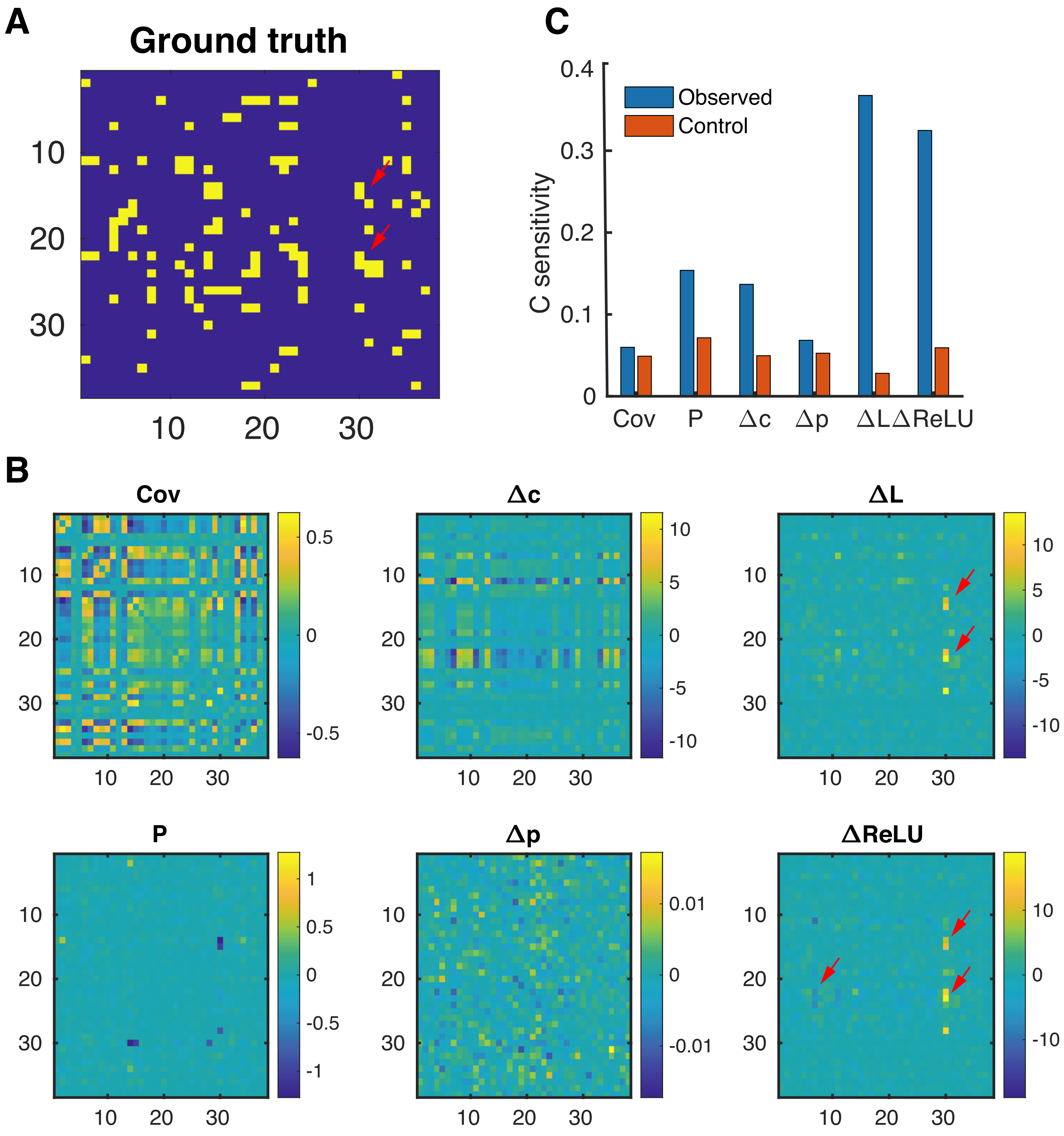

Fig. S6. DDC is sensitive to strong anatomic connections at macroscopic level (A) Intra-hemisphere strong connections (yellow entries) identified by Diffusion Spectrum Imaging). Time traces were generated using using both inter- and intra-hemisphere connections. (B) Estimated intra-hemisphere connections. Note the estimated strong connections (red arrow) in ΔL and ΔReLU correspond to true connections in (A). (C) Performance quantified by c-sensitivity. Control values were evaluated by comparing the estimation to Erdos-Renyi random graph with same sparsity.

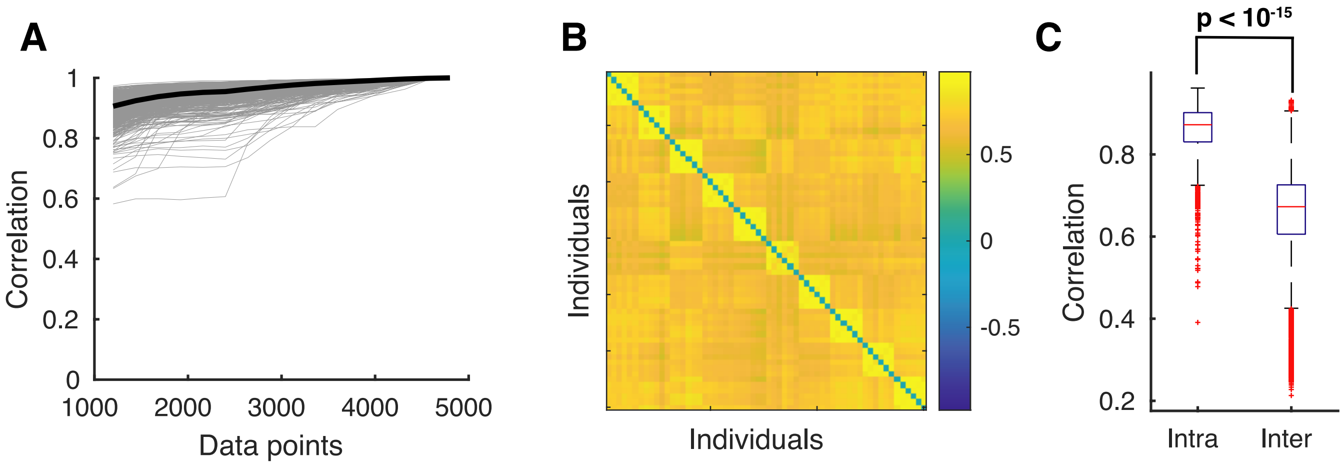

Fig. S7. Intra- and inter-individual variability of sample covariance estimation across scan session. A) Correlation of the full-length covariance estimation with covariance estimated using different amount of data. One scan session includes 1200 data points. Each gray line represents one subject and the black line is the average. B) Correlation of covariance estimated using a concatenation of two scan session within and between 10 randomly selected subjects. The block diagonal structure indicates a higher level of intra-individual similarity. C) Boxplot of intra- and inter-individual correlation pooled from all 1003 subjects (two-sided Wilcoxon rank sum test, p$< 10^{-15}$).

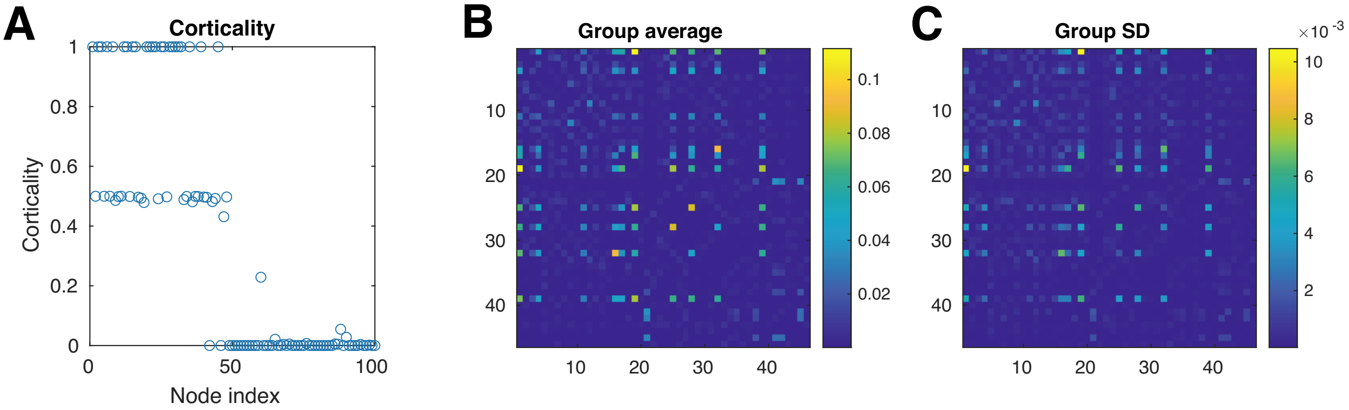

Fig. S8. Individual level dMRI statistics. (A) Corticality was defined as the proportion of cortical voxels within each IC. Since dMRI measurements are only available for cortical surface voxels. Our analysis was restricted to the first 46 ICs with corticality greater than 40%. (B) Average of the dMRI matrices across the entire 998 subjects. (C) Standard deviation.

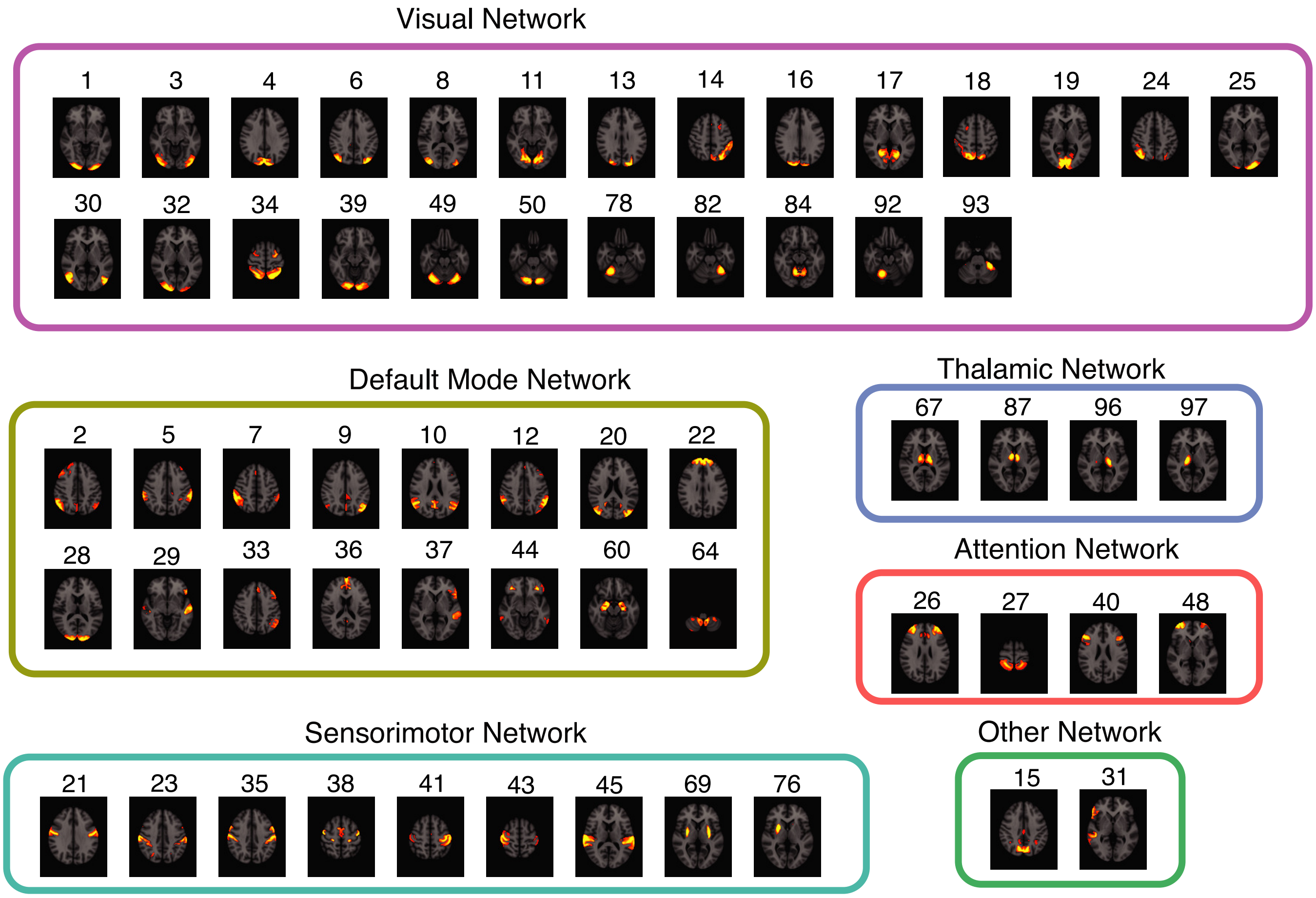

Fig. S9. Group ICA parcellation shown on an MRI template
